# Supplementary material for: NRV: An open framework for in silico evaluation of peripheral nerve electrical stimulation strategies
Source: PLoS Comput Biol. 2024 Jul 12;20(7):e1011826. doi: 10.1371/journal.pcbi.1011826 (PMC11268605; doi:10.1371/journal.pcbi.1011826)
Supplement: S1 Text — Examples of stimulus creation and combination in NRV. (PDF) [file pcbi.1011826.s001.pdf]

S1 Text: Arithmetic and logical operations between stimulus in NRV.

Arithmetic and logical operations are defined between NRV's `stimulus` class by overloading class operators. The following Python snippet shows a simple example of operations between two `stimulus` objects, `stim1` and `stim2`. The results are shown in Fig. A.

```
#nrv stimulus object declaration
stim1, stim2 = nrv.stimulus(), nrv.stimulus()

t_start = 2
duration = 10

#stim1 is a continuous squarewave
amp1 = 1
f_stim1 = 1
stim1.square(t_start, duration, amp1, f_stim1, 0, 0.5)

#stim2 is a continuous sinewave
f_stim2 = 5
amp2 = 0.5
stim2.sinus(0, t_start+duration, amp2, f_stim2)

# Arithmetic operations between stim1 and stim2
stim3 = stim1 + stim2
stim4 = stim2 - stim1
stim5 = (stim1 + 1) * stim2
```

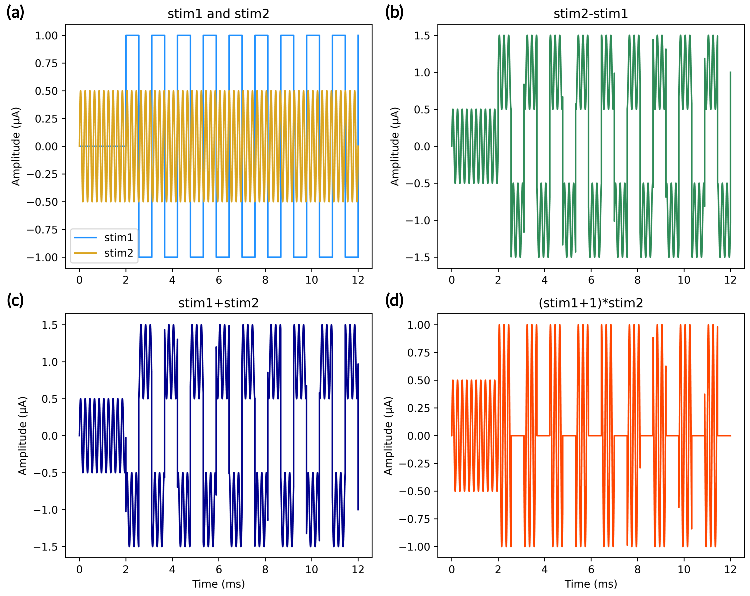

Fig A. Arithmetic operations between two stimuli in NRV.

From those operations, we can easily create more complex waveforms enabling the

evaluation of complex stimulation paradigm in NRV, as illustrated in Fig B and Fig C.

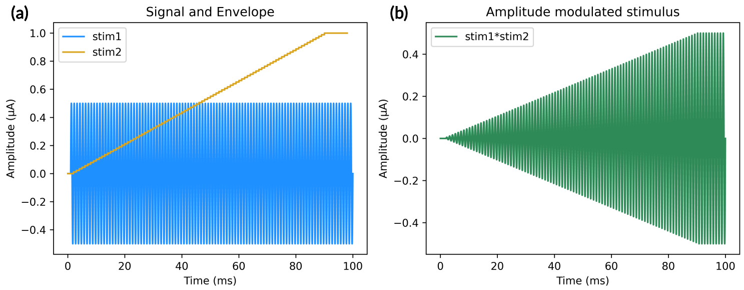

Fig B. Linearly amplitude modulated continuous sinewave.

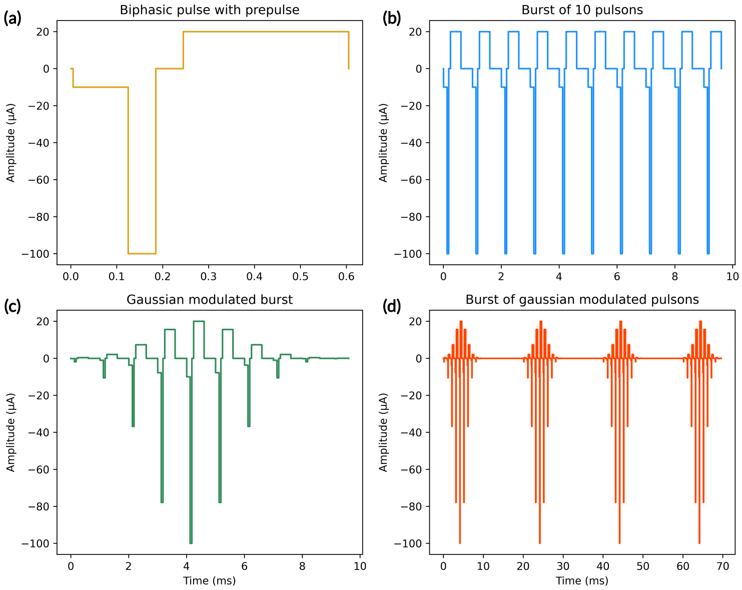

Fig C. Processus for creating a complex waveform in NRV. (a) A biphasic pulse is concatenated with a monophasic pulse to create a stimulus with a prepulse; (b) The stimulus obtained in (a) is concatenated to itself to produce a burst of 10 pulsons; (c) The burst obtained in (b) is modulated with a Gaussian waveform; (d) The Gaussian modulated burst in (c) is concatenated to itself to produce a burst of Gaussian modulated pulsons.
