# Supplementary material for: NRV: An open framework for in silico evaluation of peripheral nerve electrical stimulation strategies
Source: PLoS Comput Biol. 2024 Jul 12;20(7):e1011826. doi: 10.1371/journal.pcbi.1011826 (PMC11268605; doi:10.1371/journal.pcbi.1011826)
Supplement: S2 Text — Polynomial fit of myelinated axon structural parameters and original data. (PDF) [file pcbi.1011826.s002.pdf]

S2 Text: Myelinated axon structural parameters used in NRV.

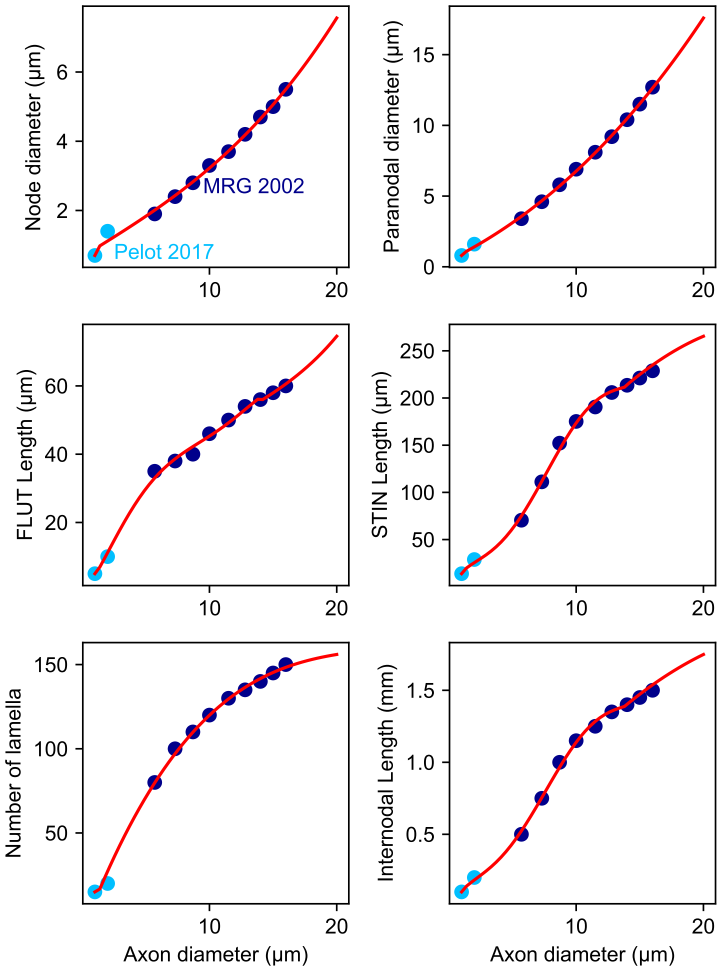

Fig A. Myelinated axon morphological parameters used in NRV. Darker blue dots are discrete fiber morphological parameters taken from the original MRG paper [43]. Lighter blue dots are morphological parameters for small myelinated axons added by Pelot *et al.* [81]. Red lines are the NRV's interpolation functions for each morphological parameter.

The interpolation equations are detailed below. In order, node diameter (d_{node}), paranodal diameter (d_{para}), FLUT length (L_{FLUT}), number of lamella ($N_{lamella}$) and internodal length (L_{inter}).

$$d_{node} = 0.0002965d_a^3 - 1.877e-05d_a^2 + 0.2266d_a + 0.6625$$

$$d_{para} = 0.0002165d_a^3 + 0.01607d_a^2 + 0.4492d_a + 0.4436$$

$$L_{FLUT} = \begin{cases} 0.01381d_a^3 - 0.5248d_a^2 + 8.908d_a - 4.17, & \text{if } (d_a < 1\mu m \text{ or } > 14\mu m) \\ -0.0005379d_a^5 + 0.02168d_a^4 - 0.2957d_a^3 \\ \qquad +1.337d_a^2 + 4.553d_a - 1.242, & \text{otherwise} \end{cases}$$

$$N_{lamella} = 0.01145d_a^3 - 0.8091d_a^2 + 19.87d_a - 9.323$$

$$L_{inter} = \begin{cases} -2.424d_a^2 + 140.1d_a - 82.9, & \text{if } (d_a < 1\mu m \text{ or } > 14\mu m) \\ 0.0185d_a^5 - 0.7269d_a^4 + 9.348d_a^3 - 42.4d_a^2 + 145.3d_a - 2.108, & \text{otherwise} \end{cases}$$
