# Supplementary material for: NRV: An open framework for in silico evaluation of peripheral nerve electrical stimulation strategies
Source: PLoS Comput Biol. 2024 Jul 12;20(7):e1011826. doi: 10.1371/journal.pcbi.1011826 (PMC11268605; doi:10.1371/journal.pcbi.1011826)
Supplement: S3 Text — Description, plot, and reference of the axon diameter distributions available in NRV. (PDF) [file pcbi.1011826.s003.pdf]

S3 Text: Axon diameter distributions available in NRV.

The statistical distributions available in NRV to generate an axon population are shown in Fig A (for myelinated fibers) and in Fig B (for non-myelinated fibers) and described below:

- `Schellens_x` is taken from human sural nerve [85]. Specifically, `Schellens_1` and `Schellens_2` are myelinated fiber distributions referring to Figure 1 and Figure 2 of [85], respectively.
- `Ochoa_x` refers to human sural nerve histology from [86]. `Ochoa_M` is derived from the myelinated fiber distribution illustrated in Figure 5b of [86]. `Ochoa_U` is derived from the unmyelinated fiber distribution illustrated in Figure 7b of [86].
- `Jacobs_x_x` refers to human sural nerve histology from [87]. Specifically, `Jacobs_9_A` and `Jacobs_9_B` are myelinated fiber distributions from a 2-week newborn (Figure 9A in [87]) and a 2-year human (Figure 9B in [87]), respectively. `Jacobs_11_A`, `Jacobs_11_B`, `Jacobs_11_C`, and `Jacobs_11_D` are unmyelinated fiber distributions taken from a 0-day (Figure 11A in [87]), 13-month (Figure 11B in [87]), 10-year (Figure 11C in [87]), and 66-year human (Figure 11D in [87]).

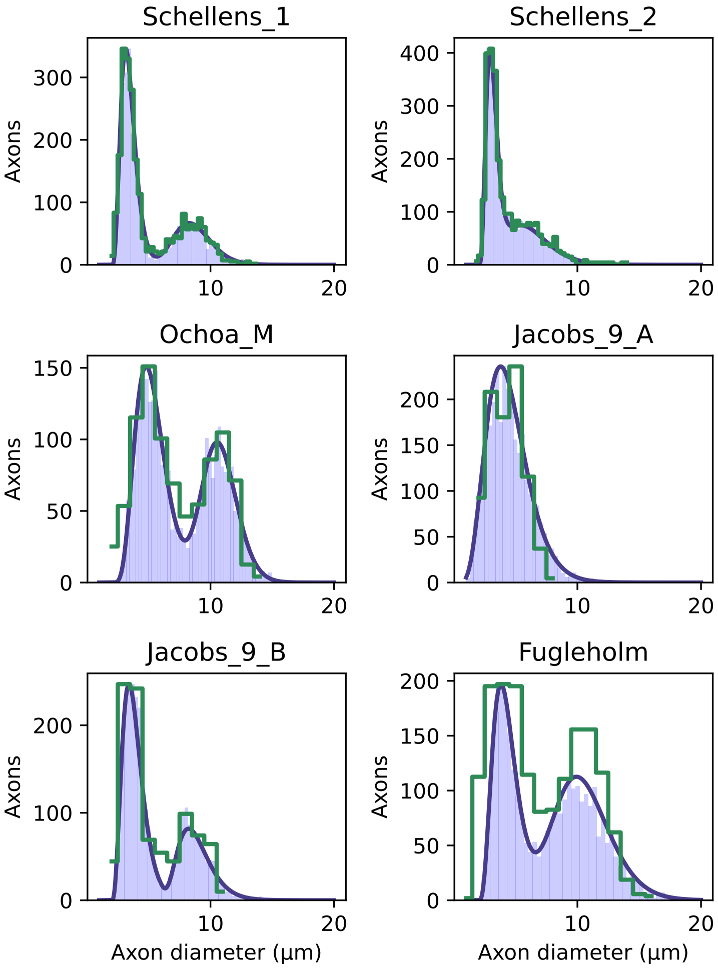

Fig A. Myelinated axon morphological parameters used in NRV. Green traces are from the original histological data; Blue traces are interpolation functions; Light blue bar plots represent a 10,000 axon population generated from the histological data.

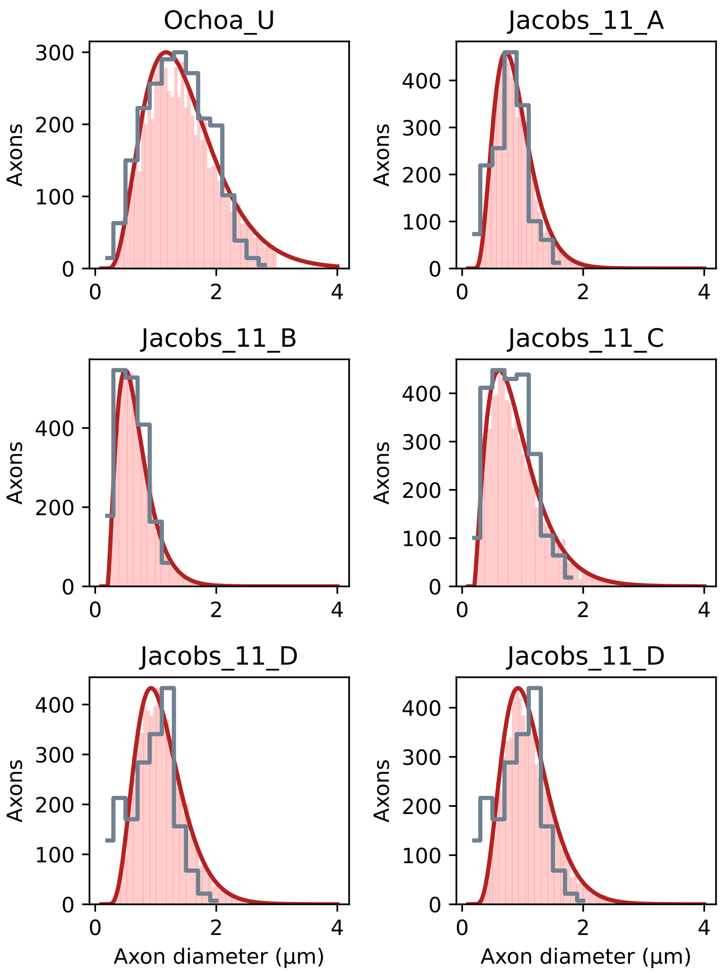

Fig B. Unmyelinated axon morphological parameters used in NRV. Grey traces are from the original histological data; Red traces are interpolation functions; Light red bar plots represent a 10,000 axon population generated from the histological data.
