# Supplementary material for: NRV: An open framework for in silico evaluation of peripheral nerve electrical stimulation strategies
Source: PLoS Comput Biol. 2024 Jul 12;20(7):e1011826. doi: 10.1371/journal.pcbi.1011826 (PMC11268605; doi:10.1371/journal.pcbi.1011826)
Supplement: S4 Text — Validation of the FEM equations and solver implementation on a 2-D bi-domain model. (PDF) [file pcbi.1011826.s004.pdf]

S4 Text: Validation of NRV's Electrical Physics Computation

The electric potential across a bi-domain 2-D box is evaluated using both the FEniCS and the COMSOL FEM solvers. The two domains Ω_1 and Ω_2 are characterized by their electrical conductivity σ_1 and σ_2 respectively. The domains are separated by a thin layer Ω_{in} of thickness ϵ_{tick} and electrical conductivity σ_{in} (Fig.A(a)). The thin layer is approximated with FEniCS with the thin-layer approximation previously described. In COMSOL, the thin layer is represented using the contact impedance boundary condition. The top and bottom of the 2-D box are insulated. The right side is grounded, and a constant current I is applied to the left side of the box.

The electric potential across the $x - axis$ of the box and relative error between COMSOL and FEniCSis estimations are shown in Fig A(b). The electric potential estimated with FEniCS matches very well with the estimation from COMSOL. The maximum relative error is about 6%, and is mainly introduced by the thin-layer approximation. Although the thin-layer approximation approach used in NRV follows the same principle as the contact impedance boundary condition of COMSOL, differences in implementation can introduce errors. Analysis suggested that COMSOL takes into account the thickness of the thin layer in the meshing of the geometry whereas the NRV implantation neglects it. The average error outside the thin layer is under 0.5%.

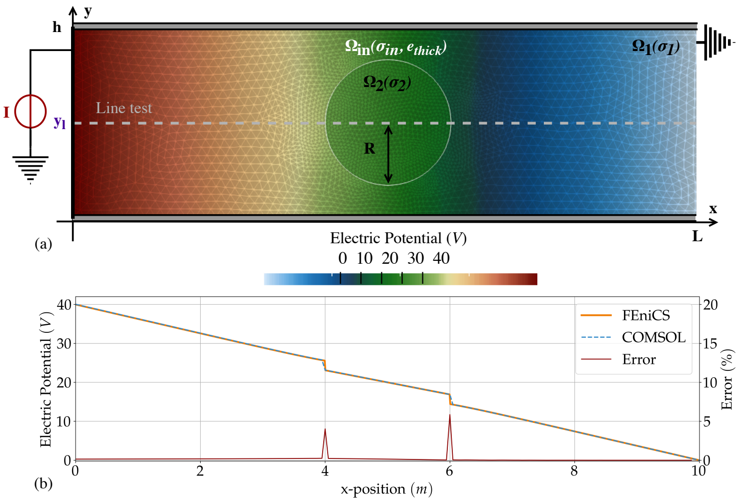

Fig A. Bi-domain 2-D box used to validate FEniCS implementation. (a) Box size is 10 by $3m$ and disk radius is $1m$. Thin-layer thickness e_{thick} is $5mm$. Domain conductivities σ_1, σ_2, and σ_{in} are respectively set to $0.9S/m$, $0.8S/m$ and $0.005S/m$. I is set to $1A$; (b) 1-D electric potential along the $x - axis$ at $y = y_1$ computed with COMSOL and FEniCs, and absolute error between the two solvers.
