# Supplementary material for: NRV: An open framework for in silico evaluation of peripheral nerve electrical stimulation strategies
Source: PLoS Comput Biol. 2024 Jul 12;20(7):e1011826. doi: 10.1371/journal.pcbi.1011826 (PMC11268605; doi:10.1371/journal.pcbi.1011826)
Supplement: S1 Table — Table of available materials in NRV with corresponding references. (PDF) [file pcbi.1011826.s009.pdf]

S1 Table: Electrical properties of materials available in NRV.

Name in NRV	Animal	Conductivity ($S.m^{-1}$)	Reference
endoneurium_ranck	Cat	0.083 (trans.)<br>0.571 (long.)	Ranck 1965 [66]
endoneurium_bhadra	NA[1]	0.2	Bhadra 2007 [31]
endoneurium_horn	Canine	1.03	Horn 2023 [68]
epineurium_horn	Canine	0.018	Horn 2023 [68]
epineurium	Canine[2]	0.085	Choi 2001 [69]
perineurium_horn	Canine	$2.7.10^{-4}$	Horn 2023 [68]
perineurium	Frog	0.002	Weerasuriya 1984 [67]

[1]The endoneurium conductivity used by Bhadra *et al.* [31] was originally defined in [70] without any reference or justification. We added it to replicate Bhadra's block studies. Use with caution. [2]The epineurium value was found in Choi *et al.* [69] is derived from the endoneurium transverse resistivity found in [66], since both materials are made of similar connective tissues. This value is often used in other *in silico* studies [24,33,34], but must be used with caution.
