# Supplementary figures and images for: NRV: An open framework for in silico evaluation of peripheral nerve electrical stimulation strategies
Source: PLoS Comput Biol. 2024 Jul 12;20(7):e1011826. doi: 10.1371/journal.pcbi.1011826 (PMC11268605; doi:10.1371/journal.pcbi.1011826)

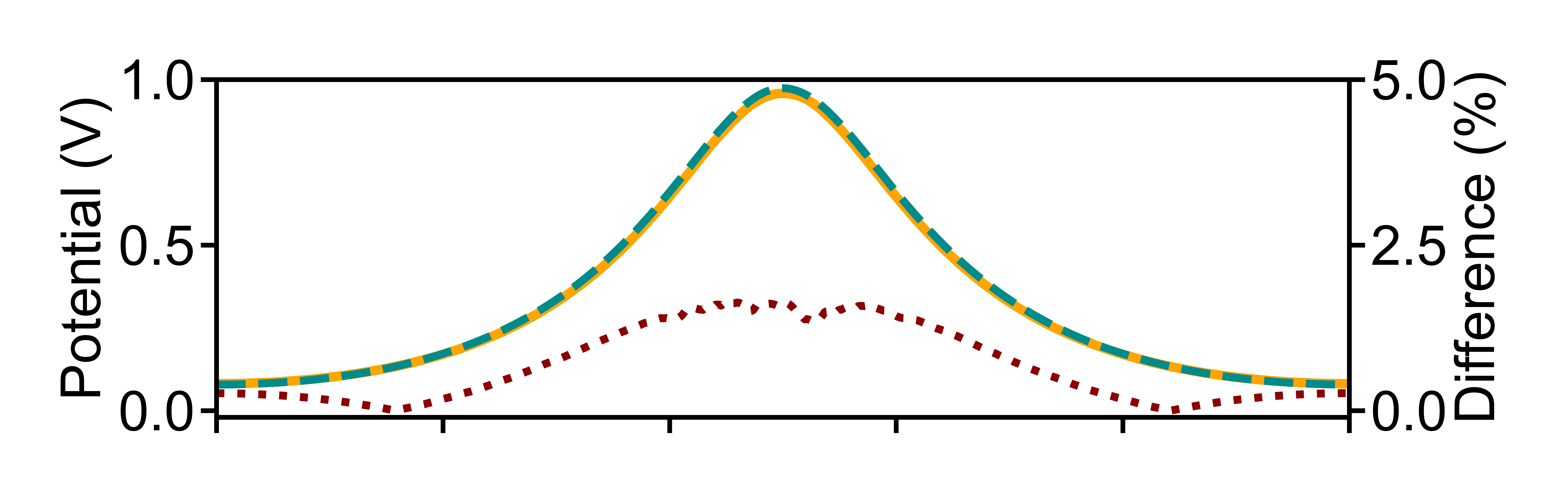

Supplement: S1 Archive — Python scripts and data files to generate and plot the comparison of the two FEM solvers. The compared data are the electrode footprint (LIFE and cuff) and the resulting activation thresholds. (ZIP) [file pcbi.1011826.s010.zip › S1_Archive/electrode_footprints/figures/2_CUFFfenxcom.png]

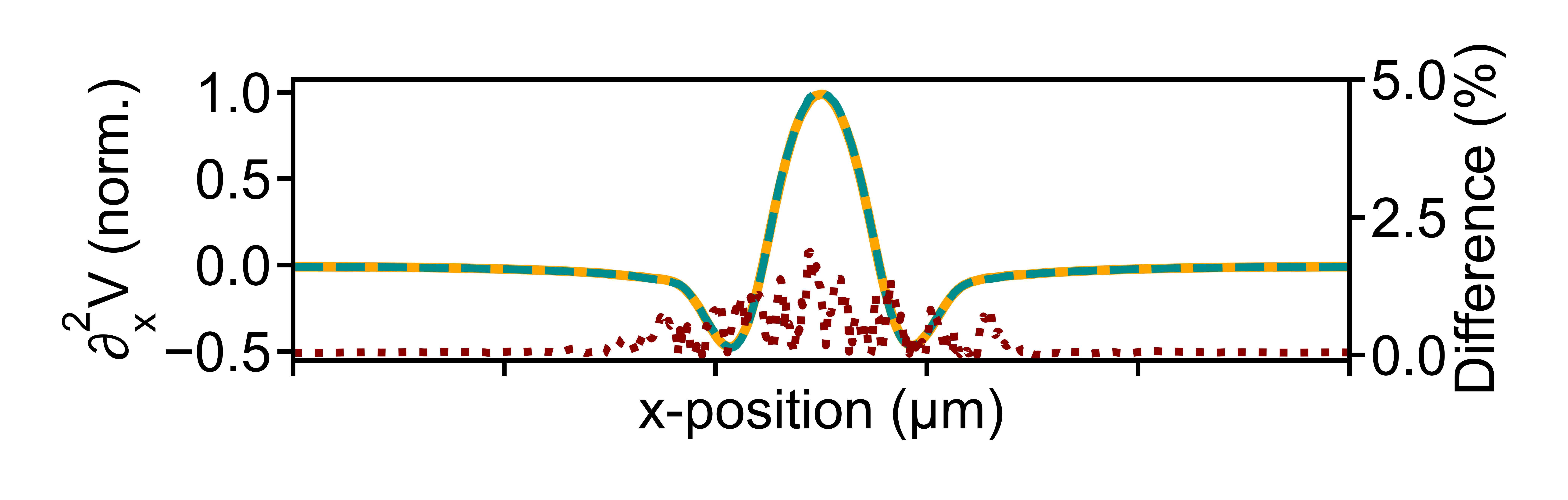

Supplement: S1 Archive — Python scripts and data files to generate and plot the comparison of the two FEM solvers. The compared data are the electrode footprint (LIFE and cuff) and the resulting activation thresholds. (ZIP) [file pcbi.1011826.s010.zip › S1_Archive/electrode_footprints/figures/2d2_LIFEfenxcom.png]

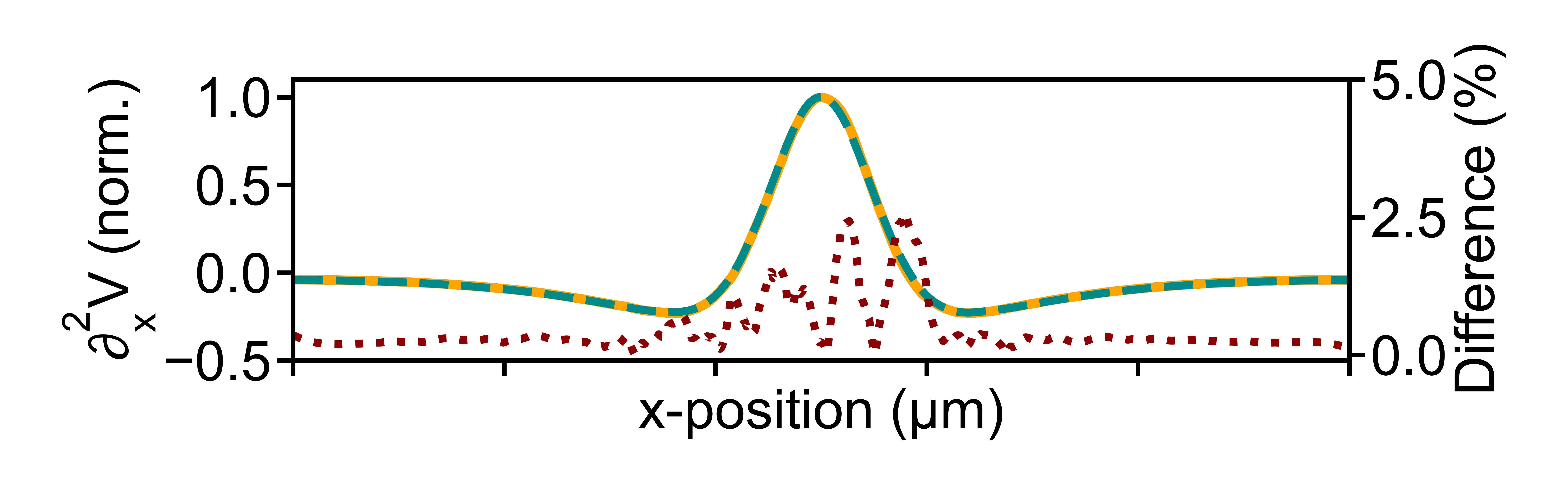

Supplement: S1 Archive — Python scripts and data files to generate and plot the comparison of the two FEM solvers. The compared data are the electrode footprint (LIFE and cuff) and the resulting activation thresholds. (ZIP) [file pcbi.1011826.s010.zip › S1_Archive/electrode_footprints/figures/2d2_CUFFfenxcom.png]

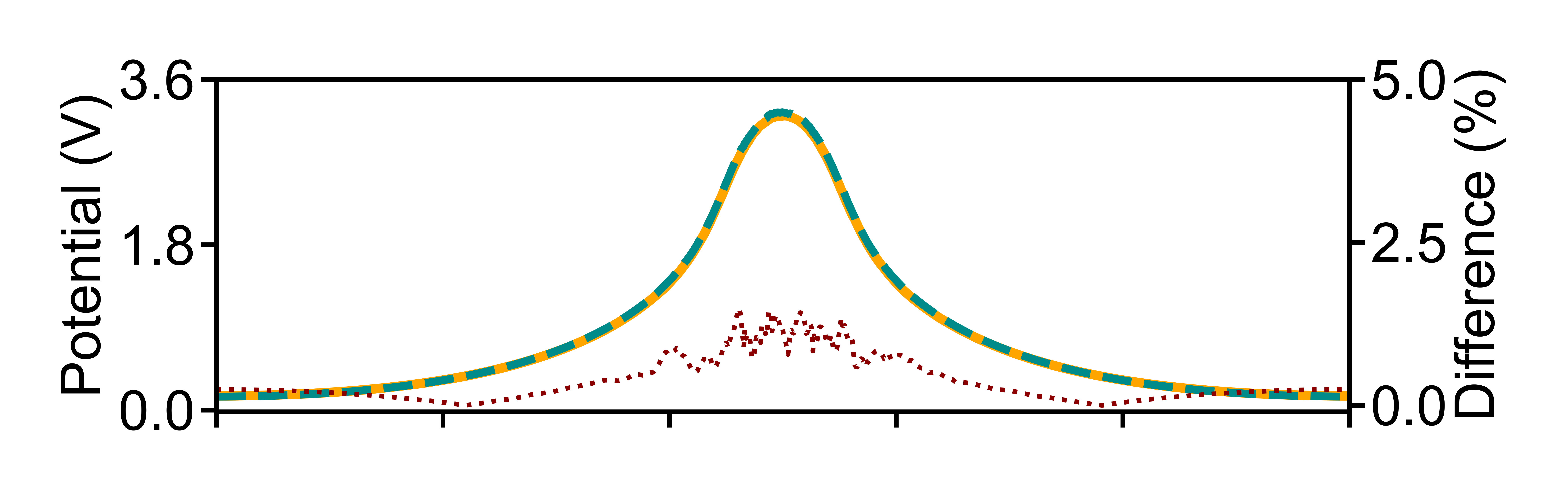

Supplement: S1 Archive — Python scripts and data files to generate and plot the comparison of the two FEM solvers. The compared data are the electrode footprint (LIFE and cuff) and the resulting activation thresholds. (ZIP) [file pcbi.1011826.s010.zip › S1_Archive/electrode_footprints/figures/2_LIFEfenxcom.png]

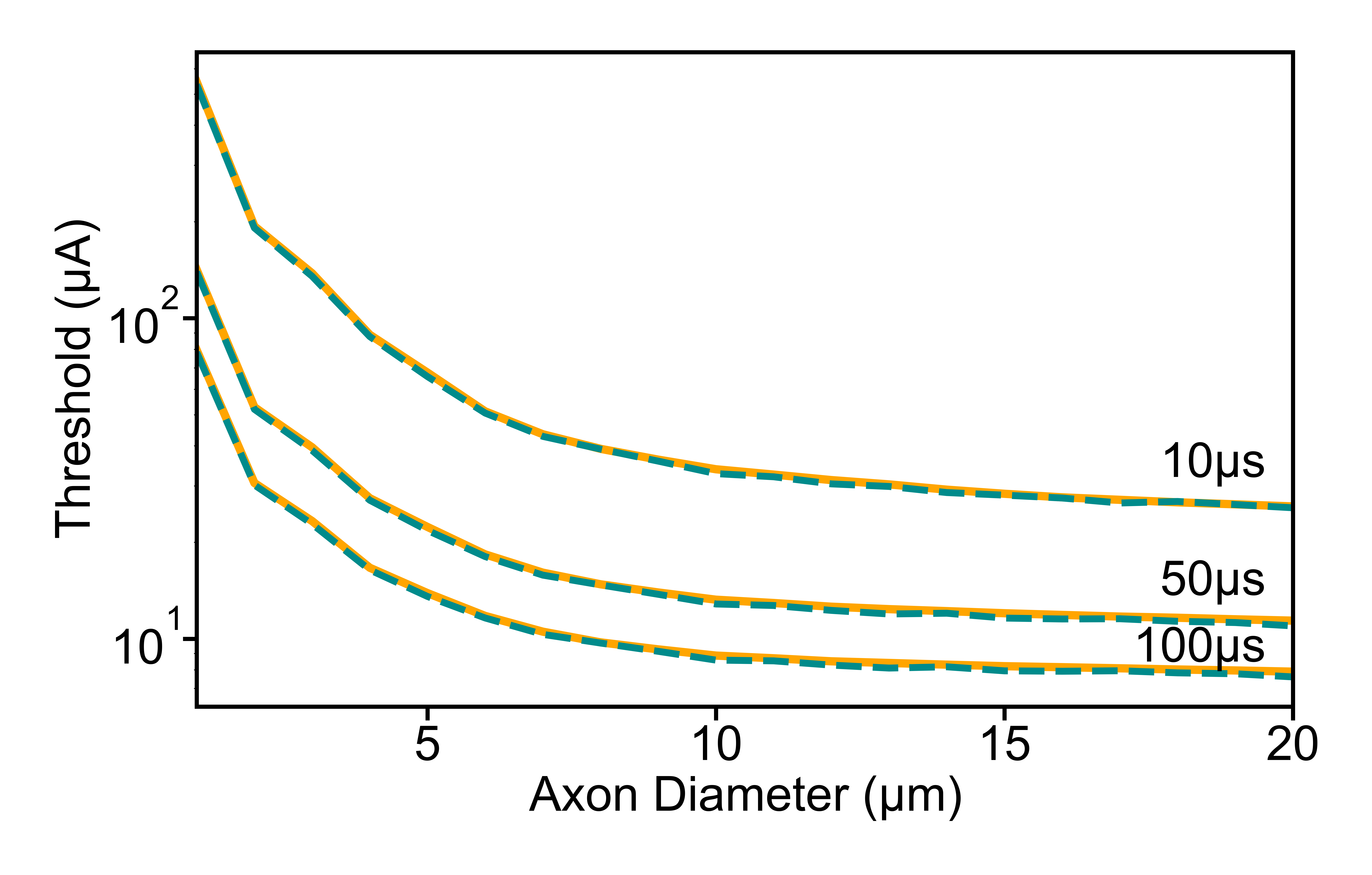

Supplement: S1 Archive — Python scripts and data files to generate and plot the comparison of the two FEM solvers. The compared data are the electrode footprint (LIFE and cuff) and the resulting activation thresholds. (ZIP) [file pcbi.1011826.s010.zip › S1_Archive/electrode_thresholds/figures/3_LIFE_thresholds.png]

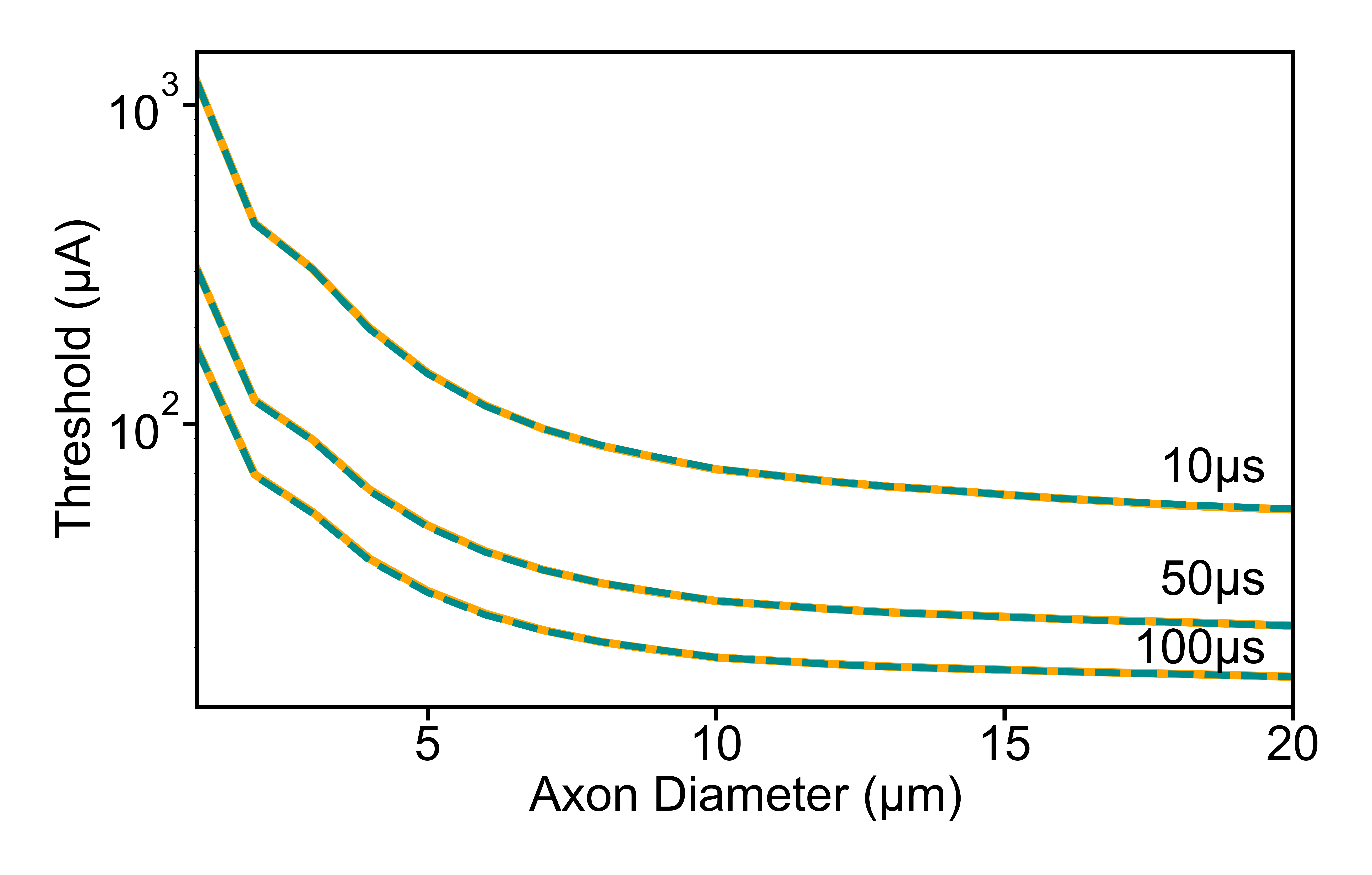

Supplement: S1 Archive — Python scripts and data files to generate and plot the comparison of the two FEM solvers. The compared data are the electrode footprint (LIFE and cuff) and the resulting activation thresholds. (ZIP) [file pcbi.1011826.s010.zip › S1_Archive/electrode_thresholds/figures/3_CUFF_thresholds.png]

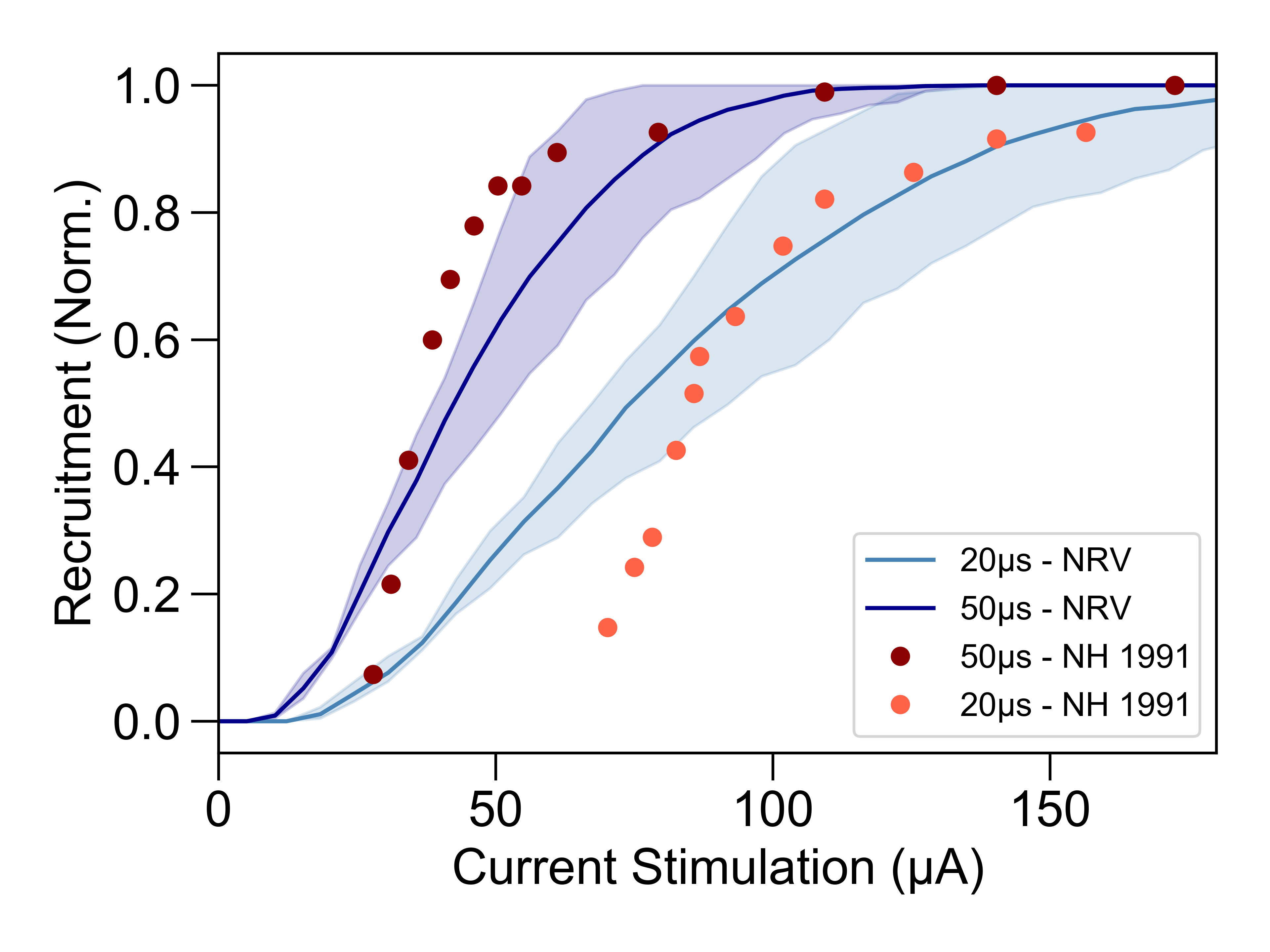

Supplement: S3 Archive — Python scripts and data files to generate and plot the in silico study replication. (ZIP) [file pcbi.1011826.s012.zip › S3_Archive/figures/Nannini_Horch_1991.png]

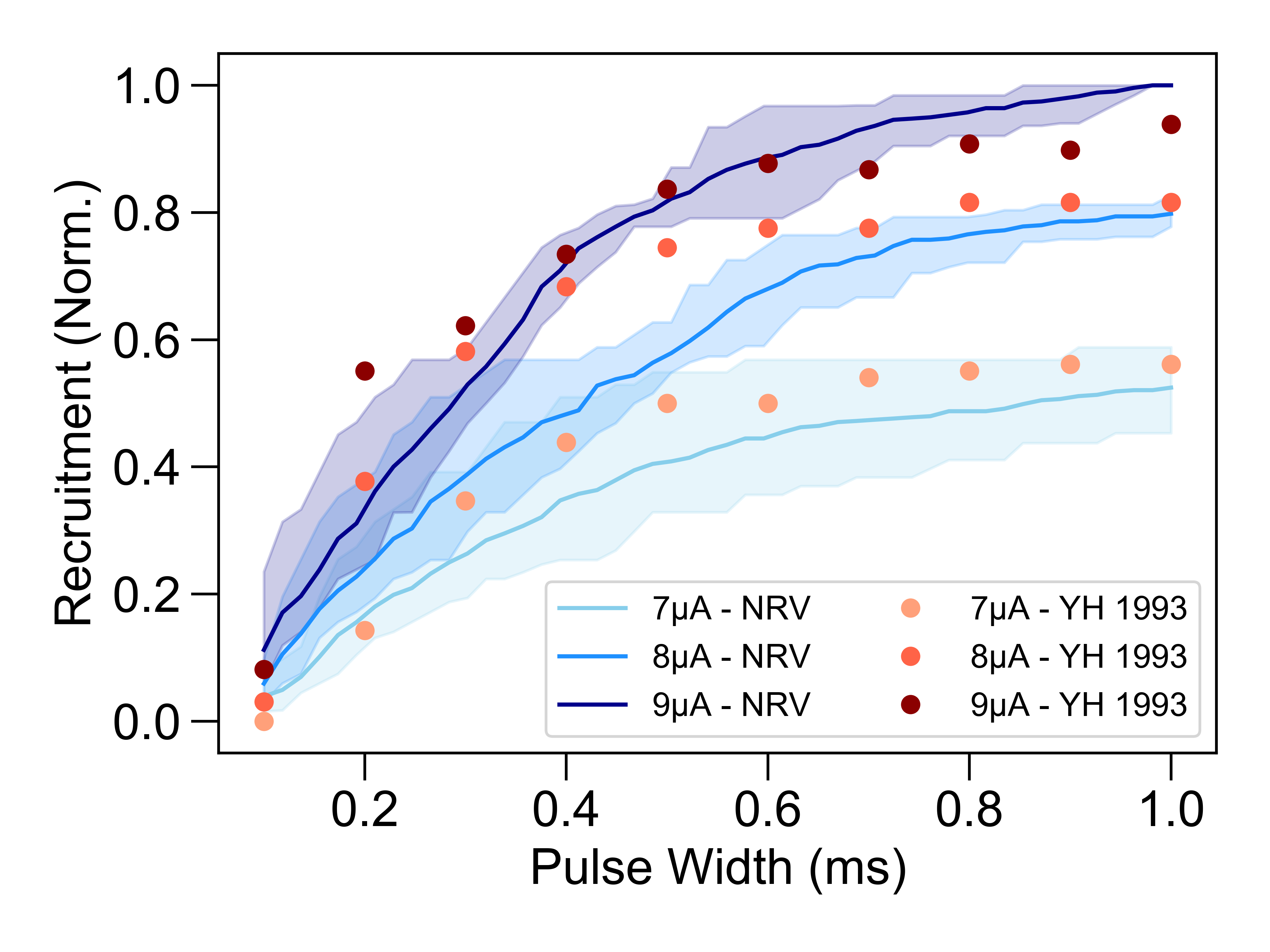

Supplement: S3 Archive — Python scripts and data files to generate and plot the in silico study replication. (ZIP) [file pcbi.1011826.s012.zip › S3_Archive/figures/Yoshida_Horch_1993.png]

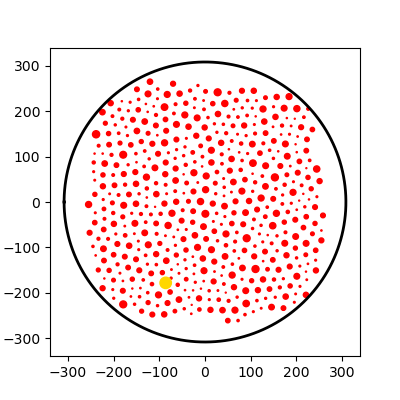

Supplement: S3 Archive — Python scripts and data files to generate and plot the in silico study replication. (ZIP) [file pcbi.1011826.s012.zip › S3_Archive/data/footprints/elec_pos_10.png]

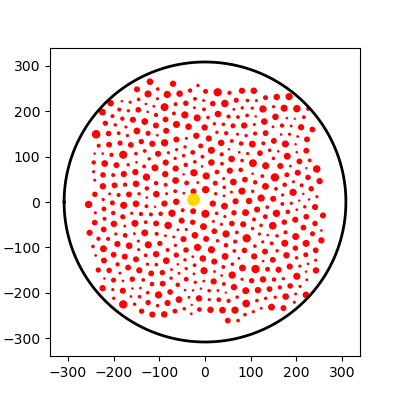

Supplement: S3 Archive — Python scripts and data files to generate and plot the in silico study replication. (ZIP) [file pcbi.1011826.s012.zip › S3_Archive/data/footprints/elec_pos_8.png]

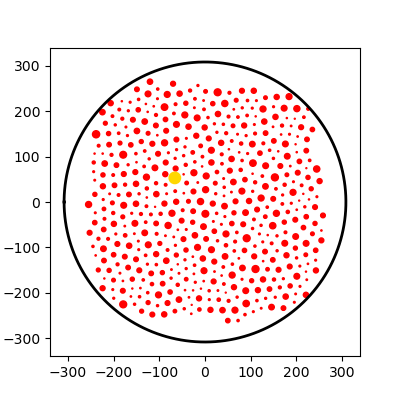

Supplement: S3 Archive — Python scripts and data files to generate and plot the in silico study replication. (ZIP) [file pcbi.1011826.s012.zip › S3_Archive/data/footprints/elec_pos_9.png]

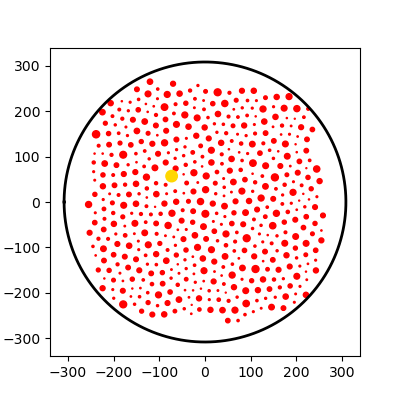

Supplement: S3 Archive — Python scripts and data files to generate and plot the in silico study replication. (ZIP) [file pcbi.1011826.s012.zip › S3_Archive/data/footprints/elec_pos_4.png]

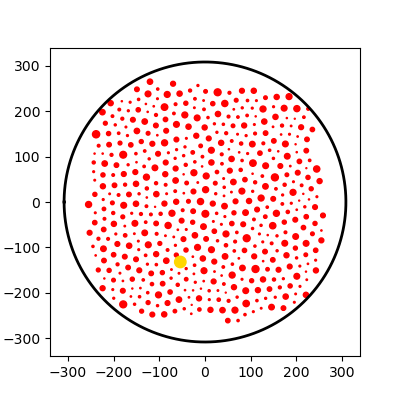

Supplement: S3 Archive — Python scripts and data files to generate and plot the in silico study replication. (ZIP) [file pcbi.1011826.s012.zip › S3_Archive/data/footprints/elec_pos_5.png]

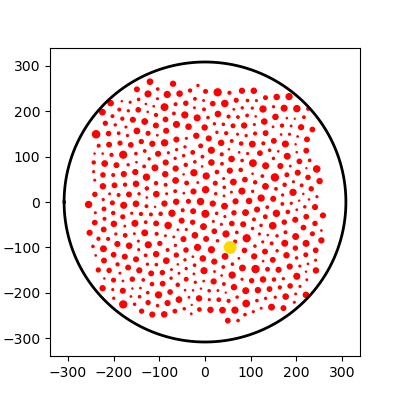

Supplement: S3 Archive — Python scripts and data files to generate and plot the in silico study replication. (ZIP) [file pcbi.1011826.s012.zip › S3_Archive/data/footprints/elec_pos_7.png]

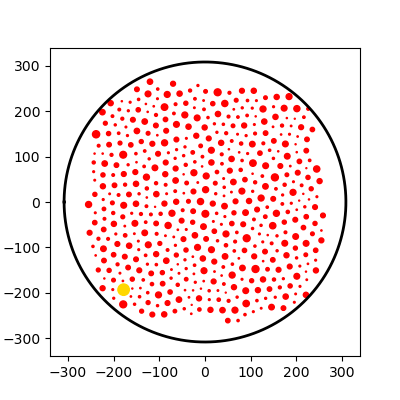

Supplement: S3 Archive — Python scripts and data files to generate and plot the in silico study replication. (ZIP) [file pcbi.1011826.s012.zip › S3_Archive/data/footprints/elec_pos_6.png]

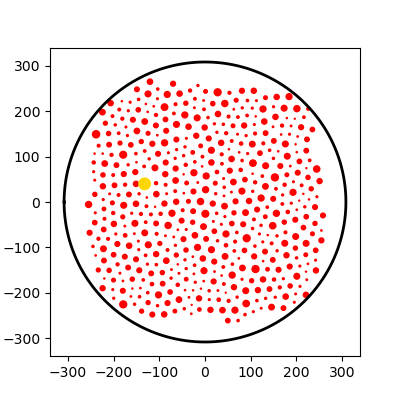

Supplement: S3 Archive — Python scripts and data files to generate and plot the in silico study replication. (ZIP) [file pcbi.1011826.s012.zip › S3_Archive/data/footprints/elec_pos_2.png]

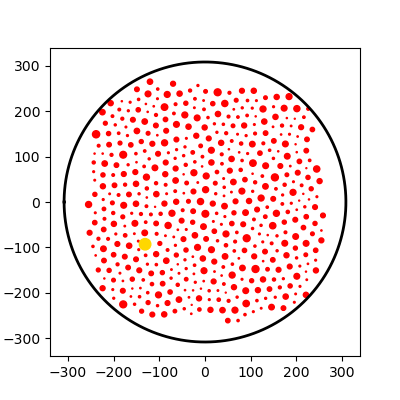

Supplement: S3 Archive — Python scripts and data files to generate and plot the in silico study replication. (ZIP) [file pcbi.1011826.s012.zip › S3_Archive/data/footprints/elec_pos_3.png]

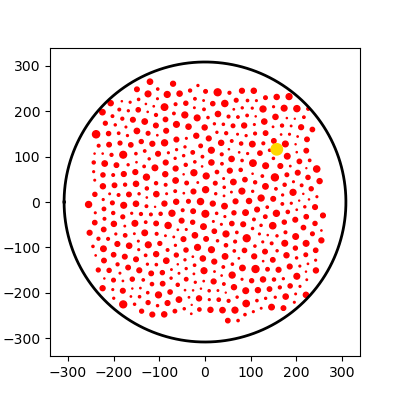

Supplement: S3 Archive — Python scripts and data files to generate and plot the in silico study replication. (ZIP) [file pcbi.1011826.s012.zip › S3_Archive/data/footprints/elec_pos_1.png]

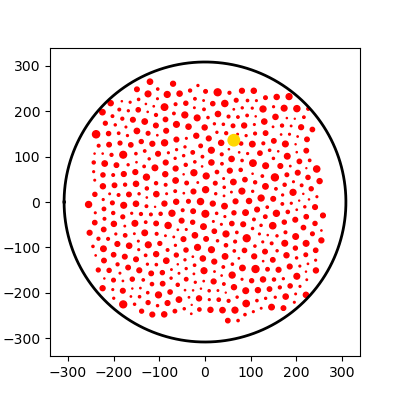

Supplement: S3 Archive — Python scripts and data files to generate and plot the in silico study replication. (ZIP) [file pcbi.1011826.s012.zip › S3_Archive/data/footprints/elec_pos_0.png]

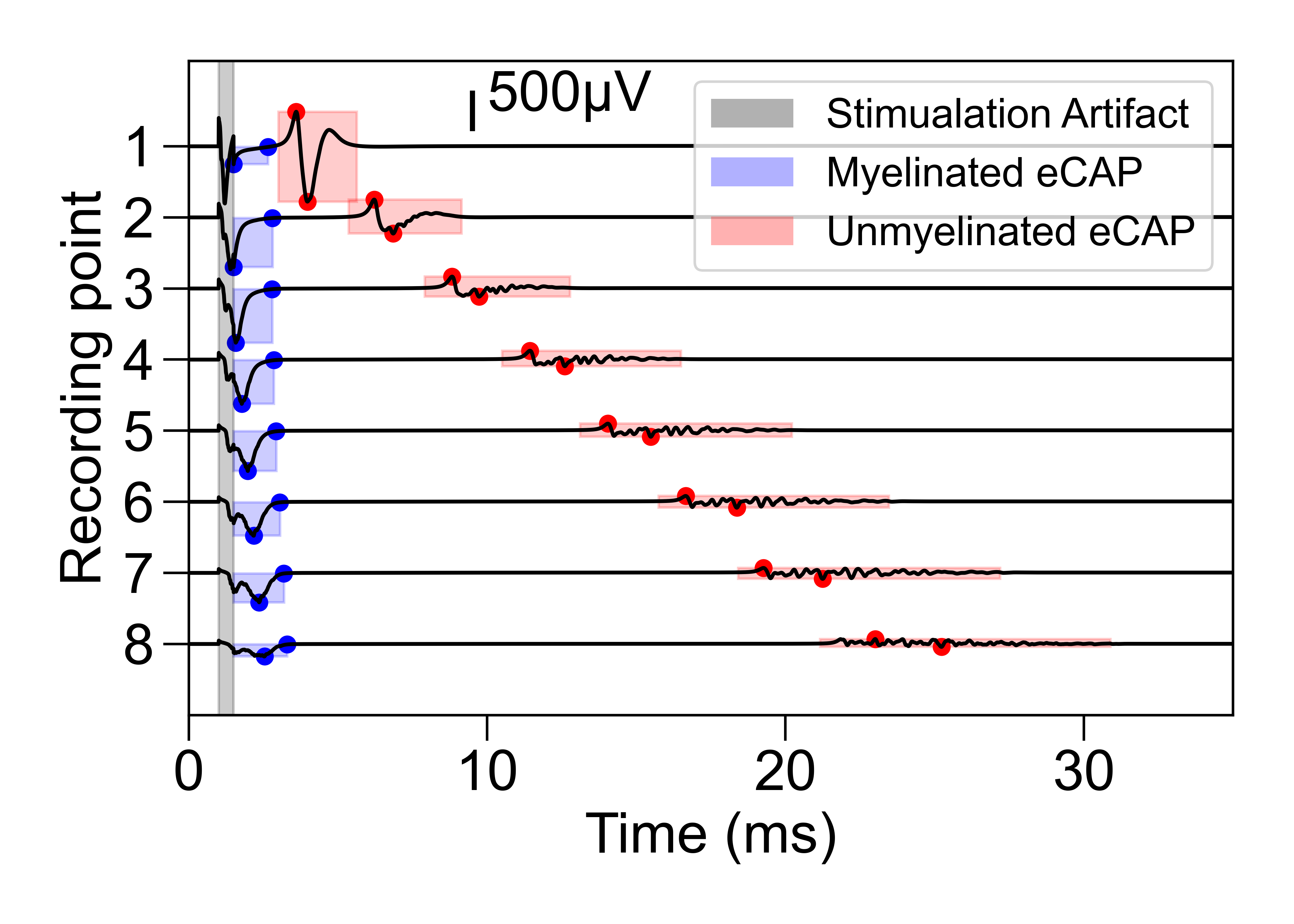

Supplement: S4 Archive — Python scripts and data files to generate and plot the in silico extracellular study. (ZIP) [file pcbi.1011826.s013.zip › S4_Archive/figures/recordings_200_3pp.png]

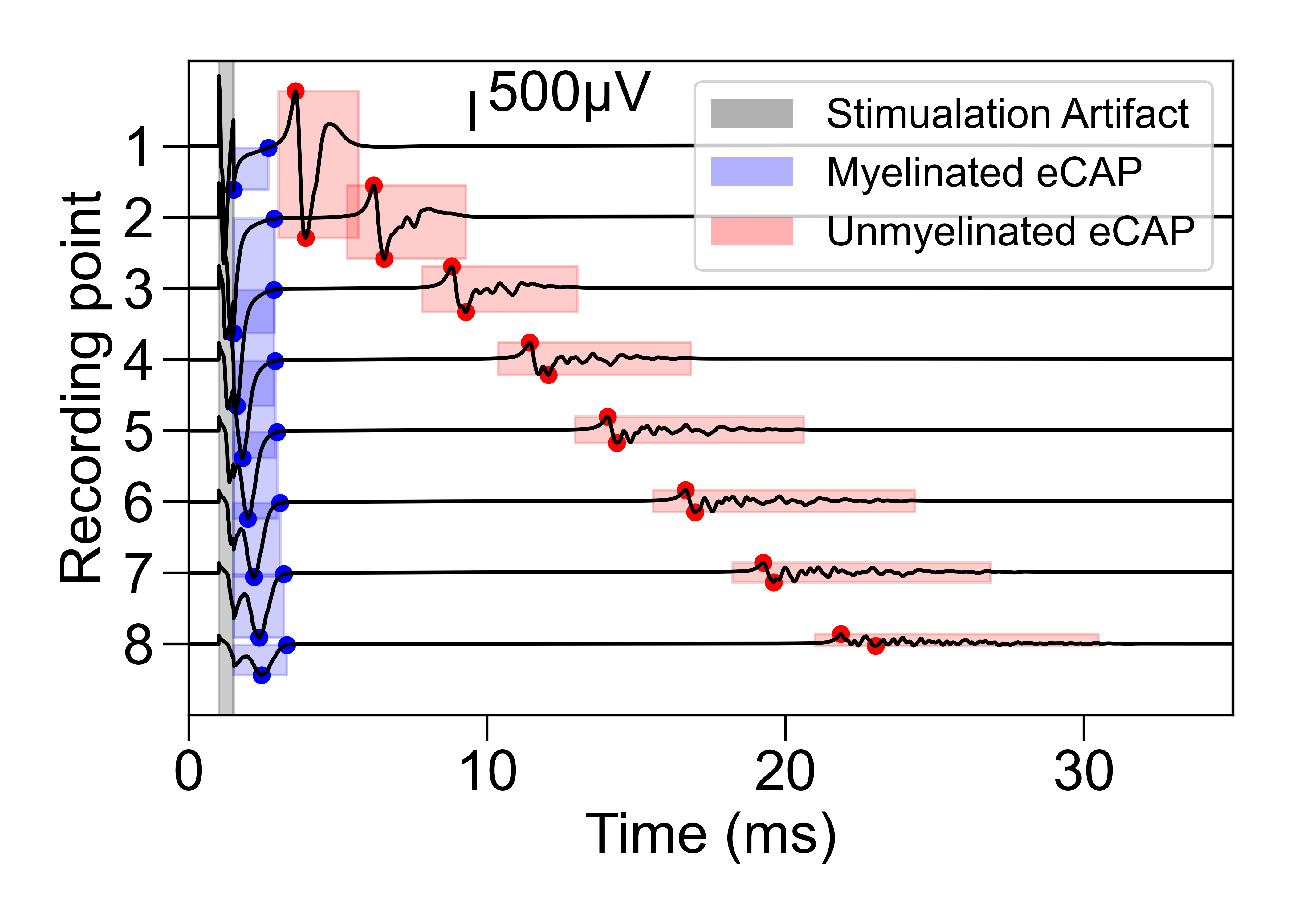

Supplement: S4 Archive — Python scripts and data files to generate and plot the in silico extracellular study. (ZIP) [file pcbi.1011826.s013.zip › S4_Archive/figures/recordings_500_4pp.png]

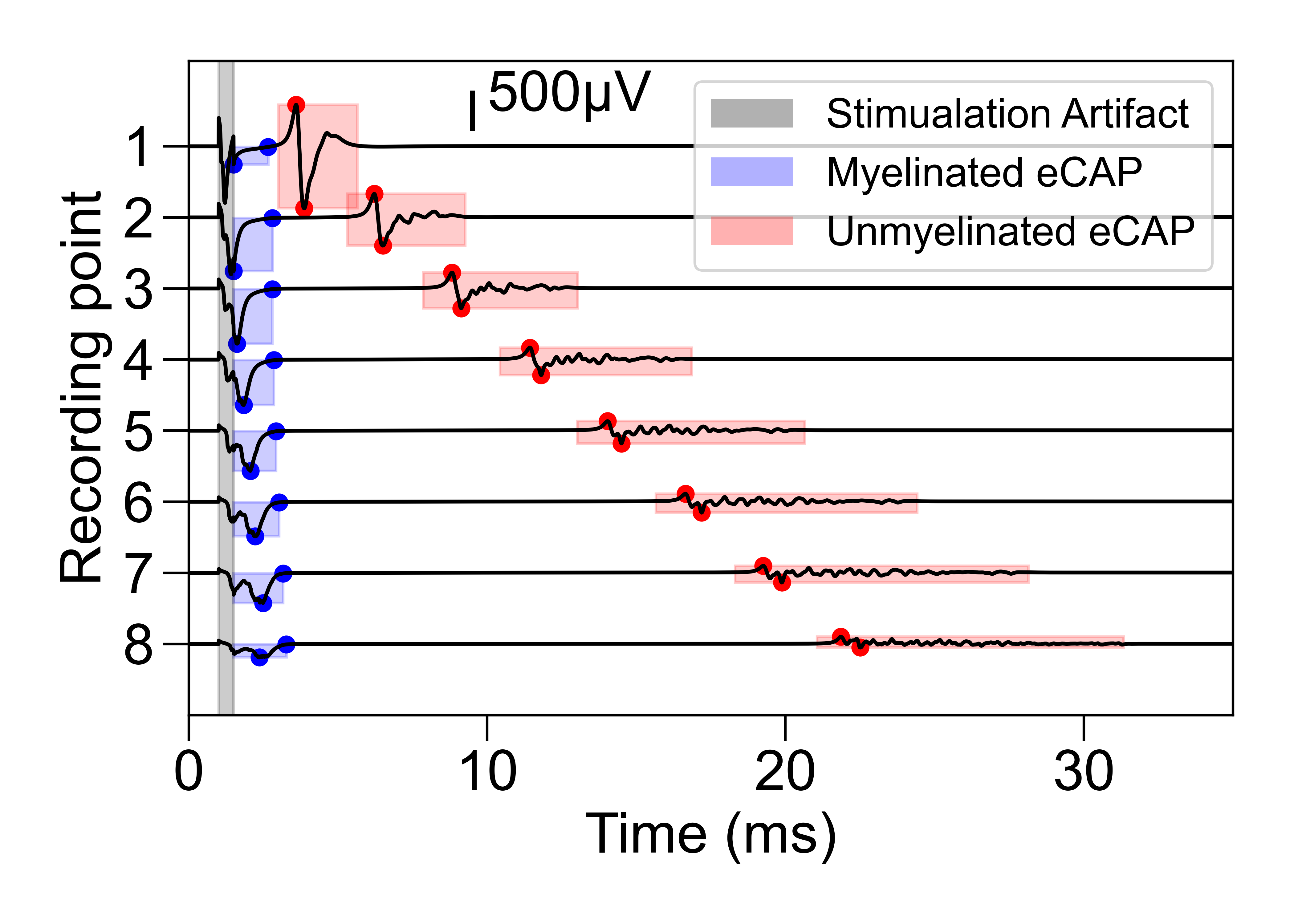

Supplement: S4 Archive — Python scripts and data files to generate and plot the in silico extracellular study. (ZIP) [file pcbi.1011826.s013.zip › S4_Archive/figures/recordings_200_1pp.png]

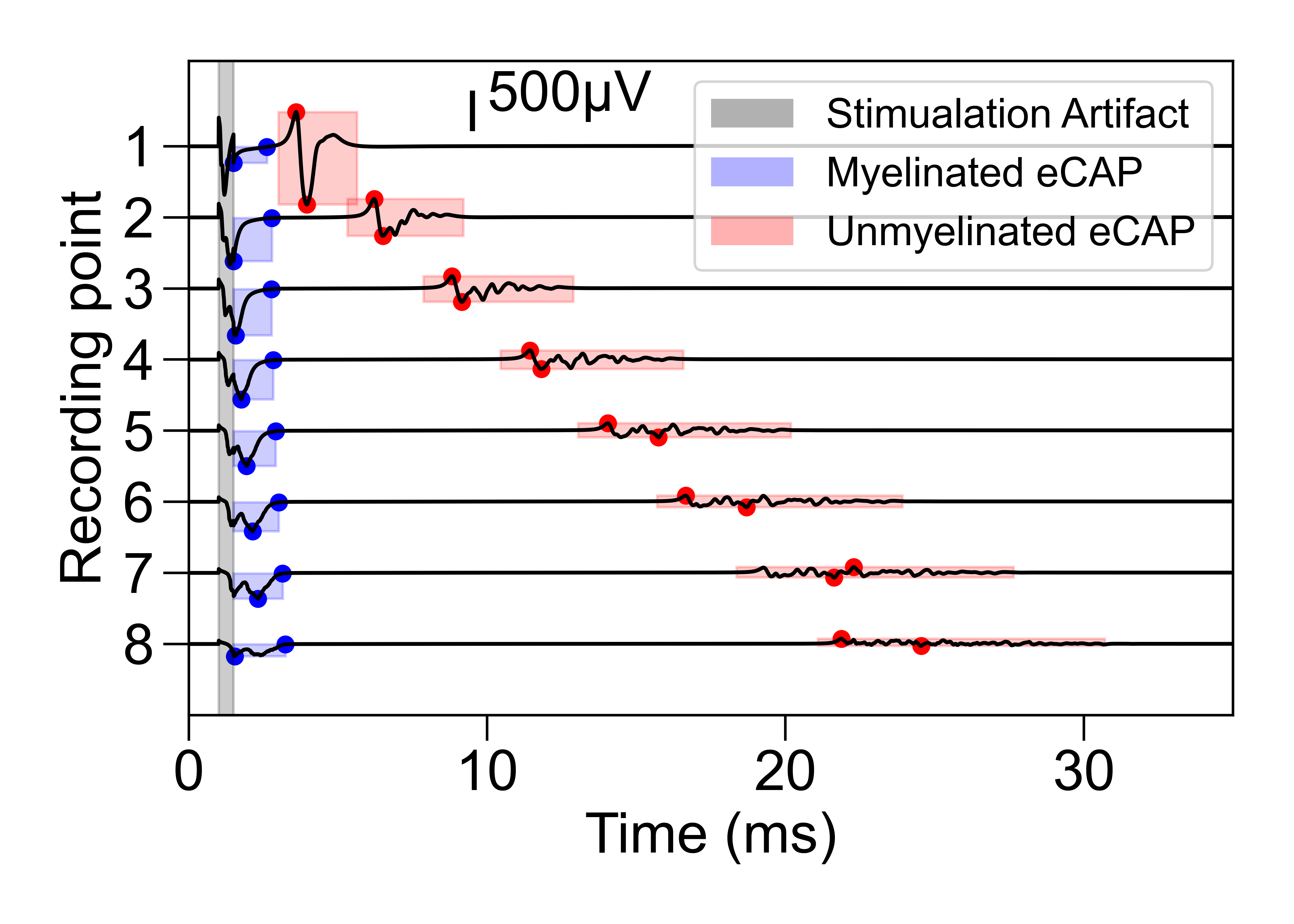

Supplement: S4 Archive — Python scripts and data files to generate and plot the in silico extracellular study. (ZIP) [file pcbi.1011826.s013.zip › S4_Archive/figures/recordings_200_5pp.png]

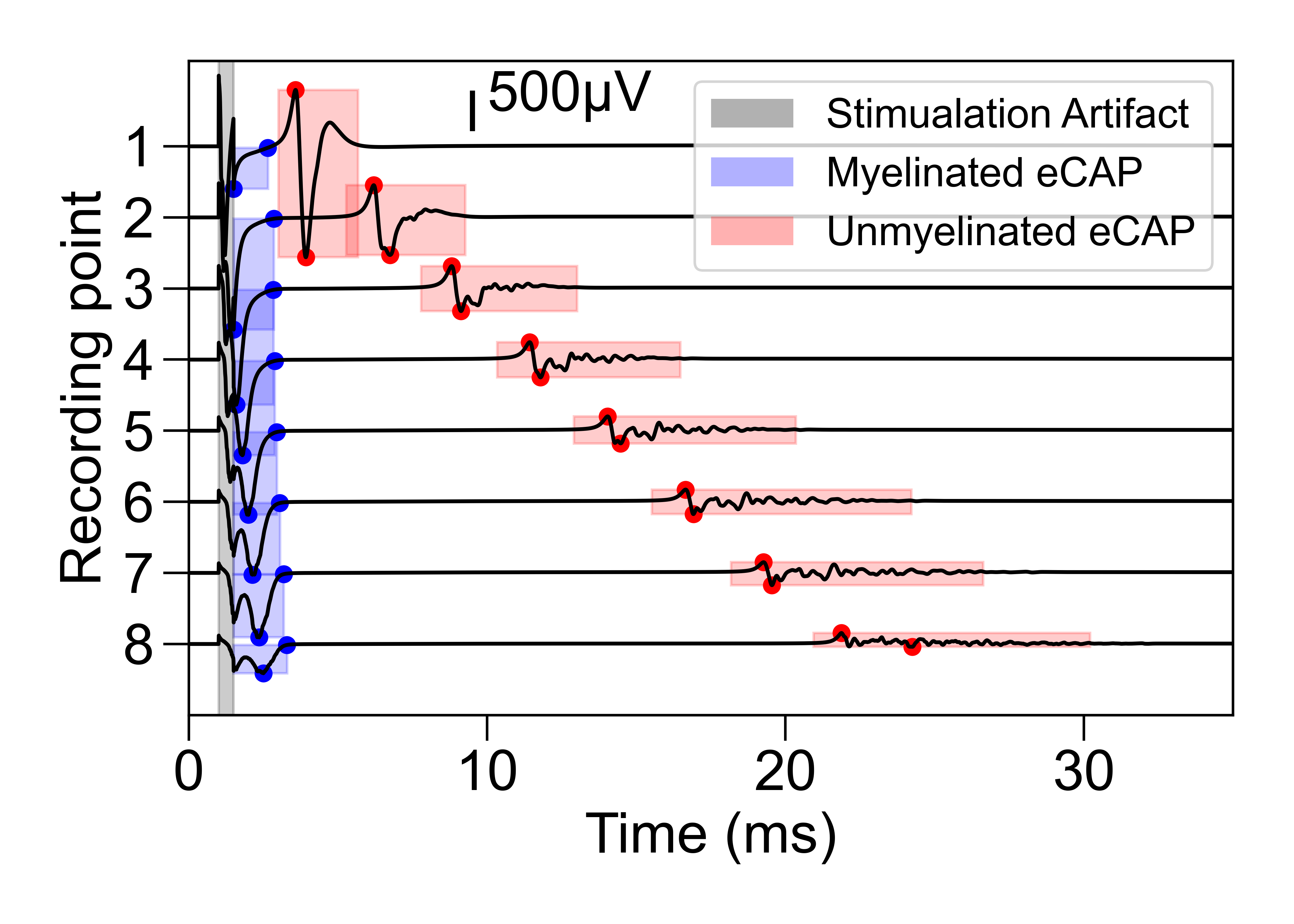

Supplement: S4 Archive — Python scripts and data files to generate and plot the in silico extracellular study. (ZIP) [file pcbi.1011826.s013.zip › S4_Archive/figures/recordings_500_2pp.png]

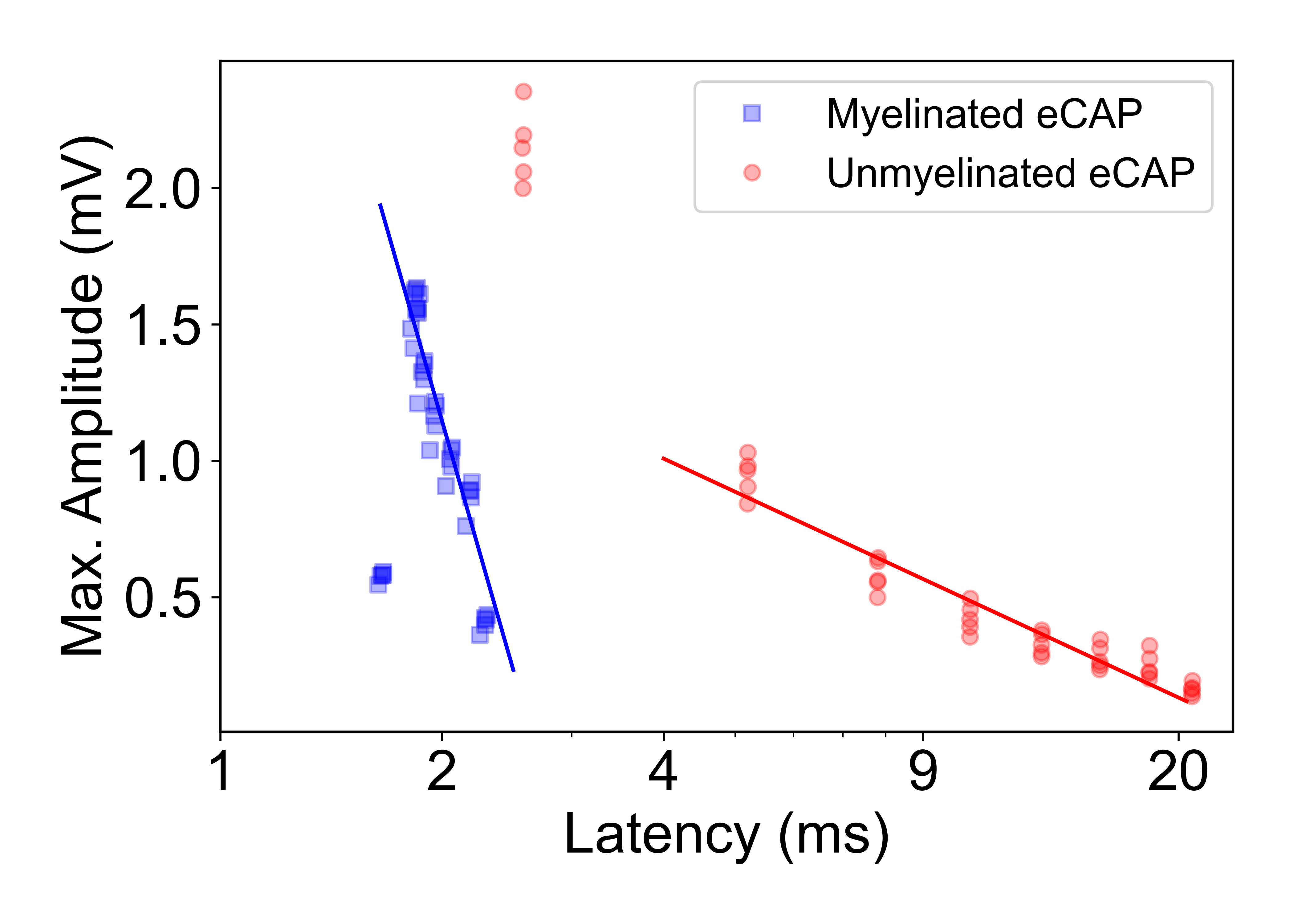

Supplement: S4 Archive — Python scripts and data files to generate and plot the in silico extracellular study. (ZIP) [file pcbi.1011826.s013.zip › S4_Archive/figures/Amplitude_latency_plot.png]

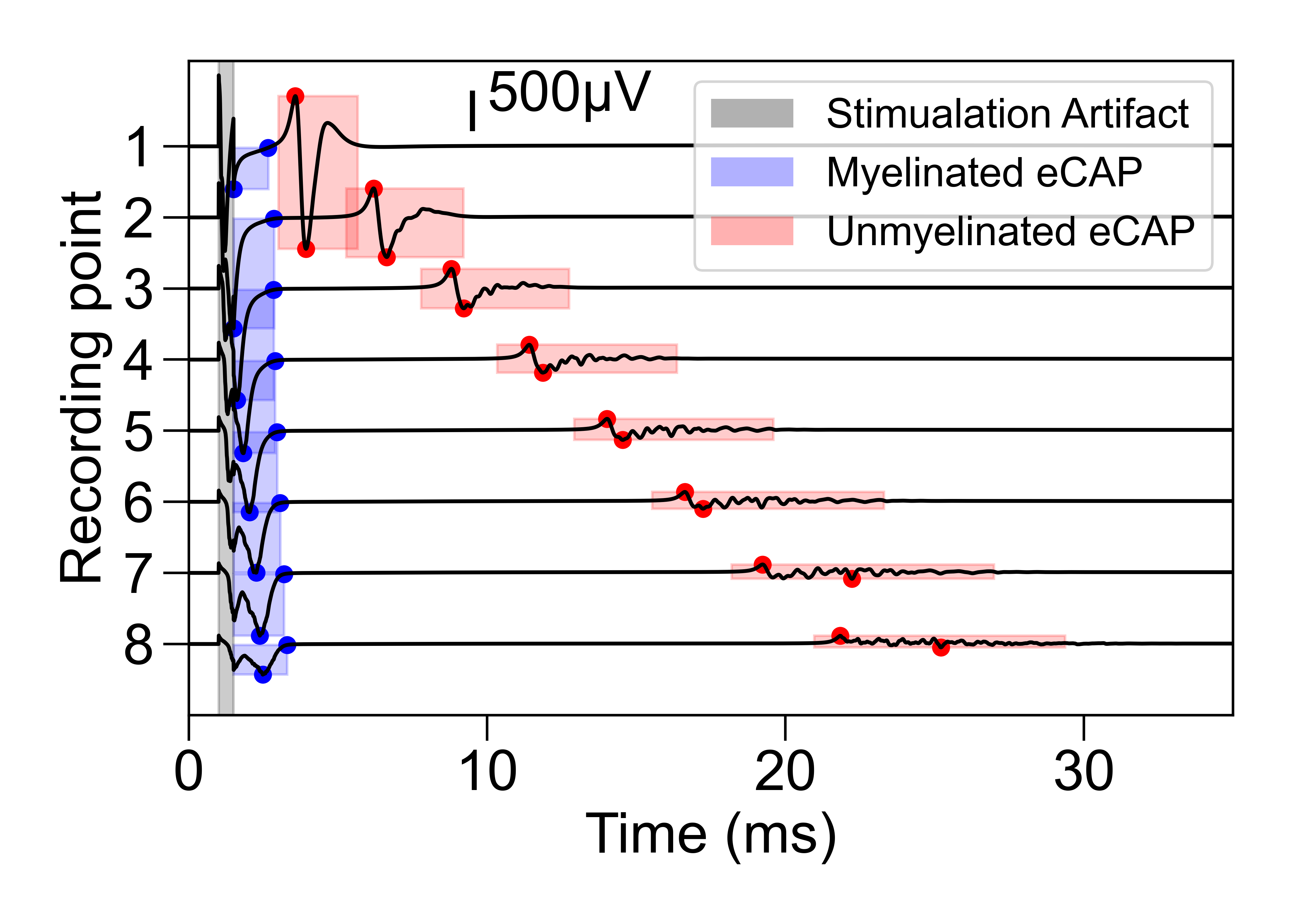

Supplement: S4 Archive — Python scripts and data files to generate and plot the in silico extracellular study. (ZIP) [file pcbi.1011826.s013.zip › S4_Archive/figures/recordings_500_5pp.png]

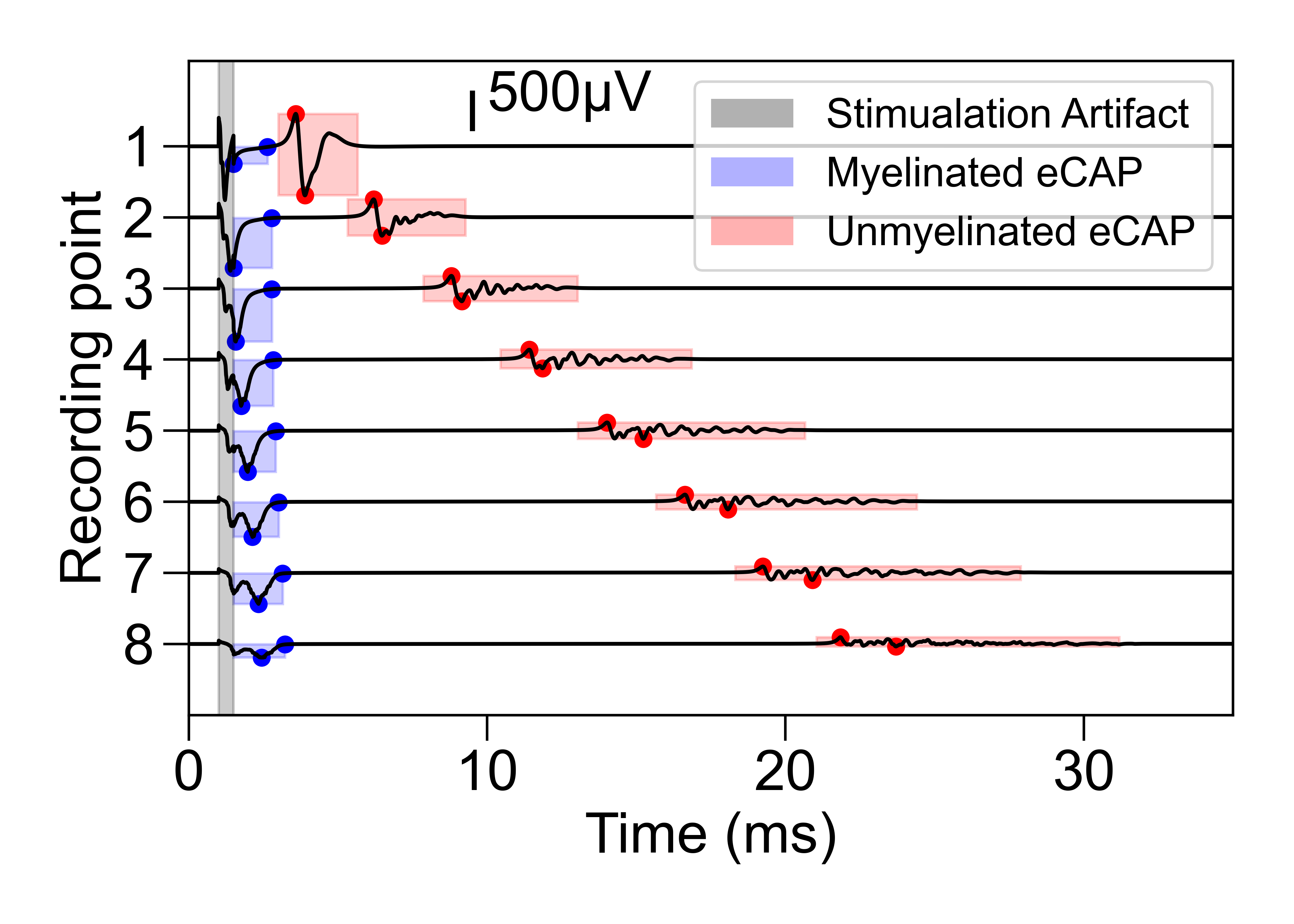

Supplement: S4 Archive — Python scripts and data files to generate and plot the in silico extracellular study. (ZIP) [file pcbi.1011826.s013.zip › S4_Archive/figures/recordings_200_2pp.png]

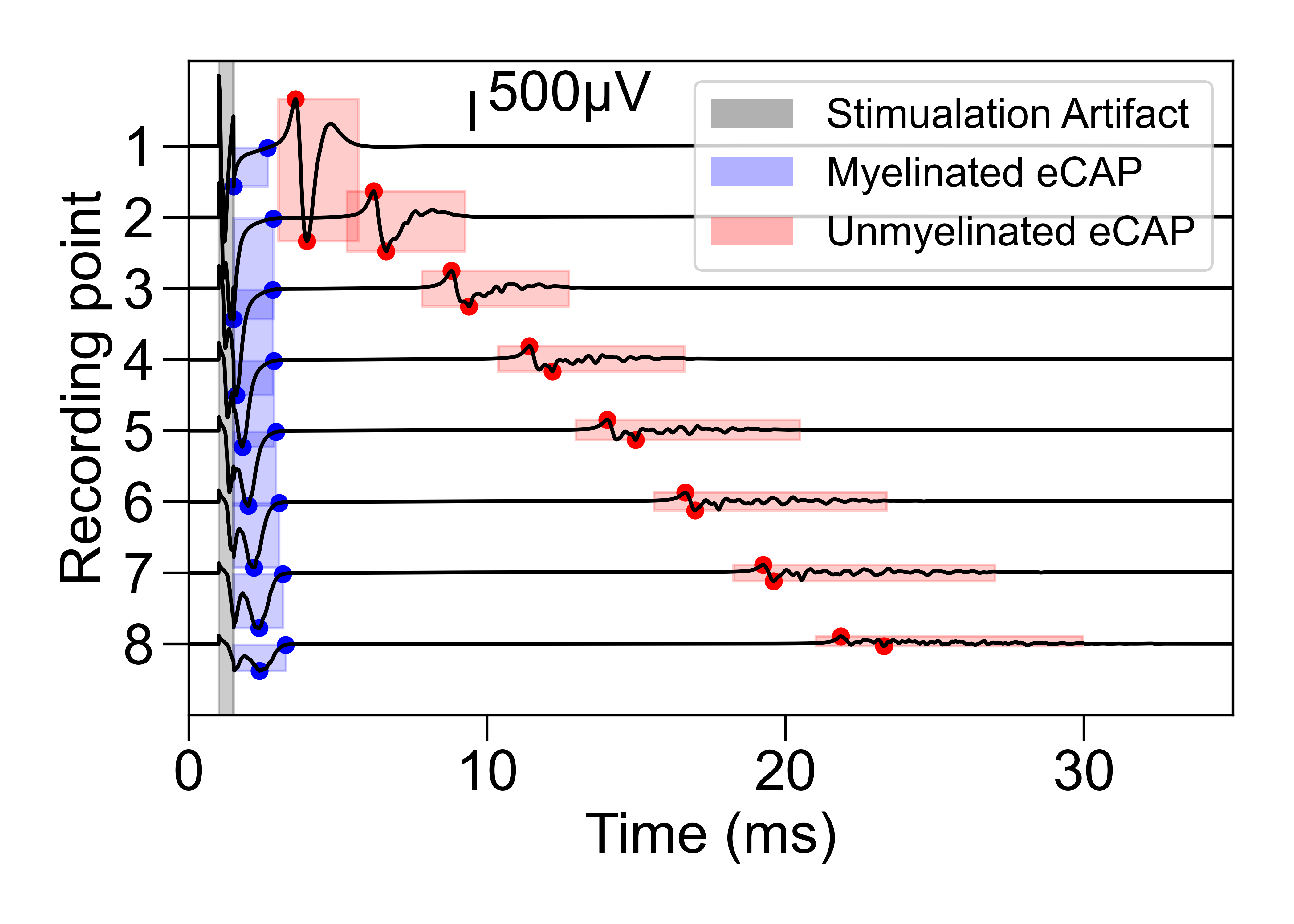

Supplement: S4 Archive — Python scripts and data files to generate and plot the in silico extracellular study. (ZIP) [file pcbi.1011826.s013.zip › S4_Archive/figures/recordings_500_3pp.png]

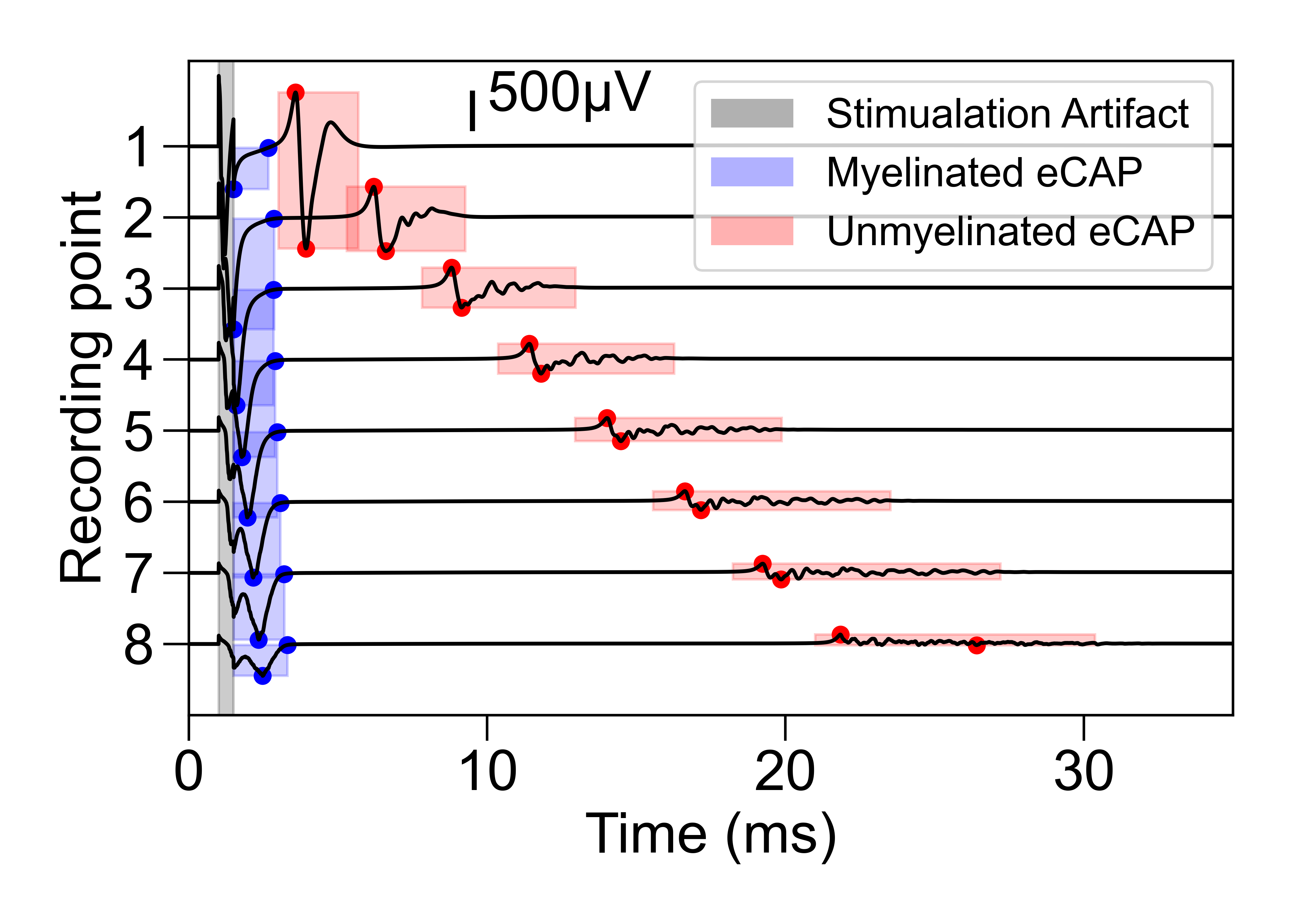

Supplement: S4 Archive — Python scripts and data files to generate and plot the in silico extracellular study. (ZIP) [file pcbi.1011826.s013.zip › S4_Archive/figures/recordings_500_1pp.png]

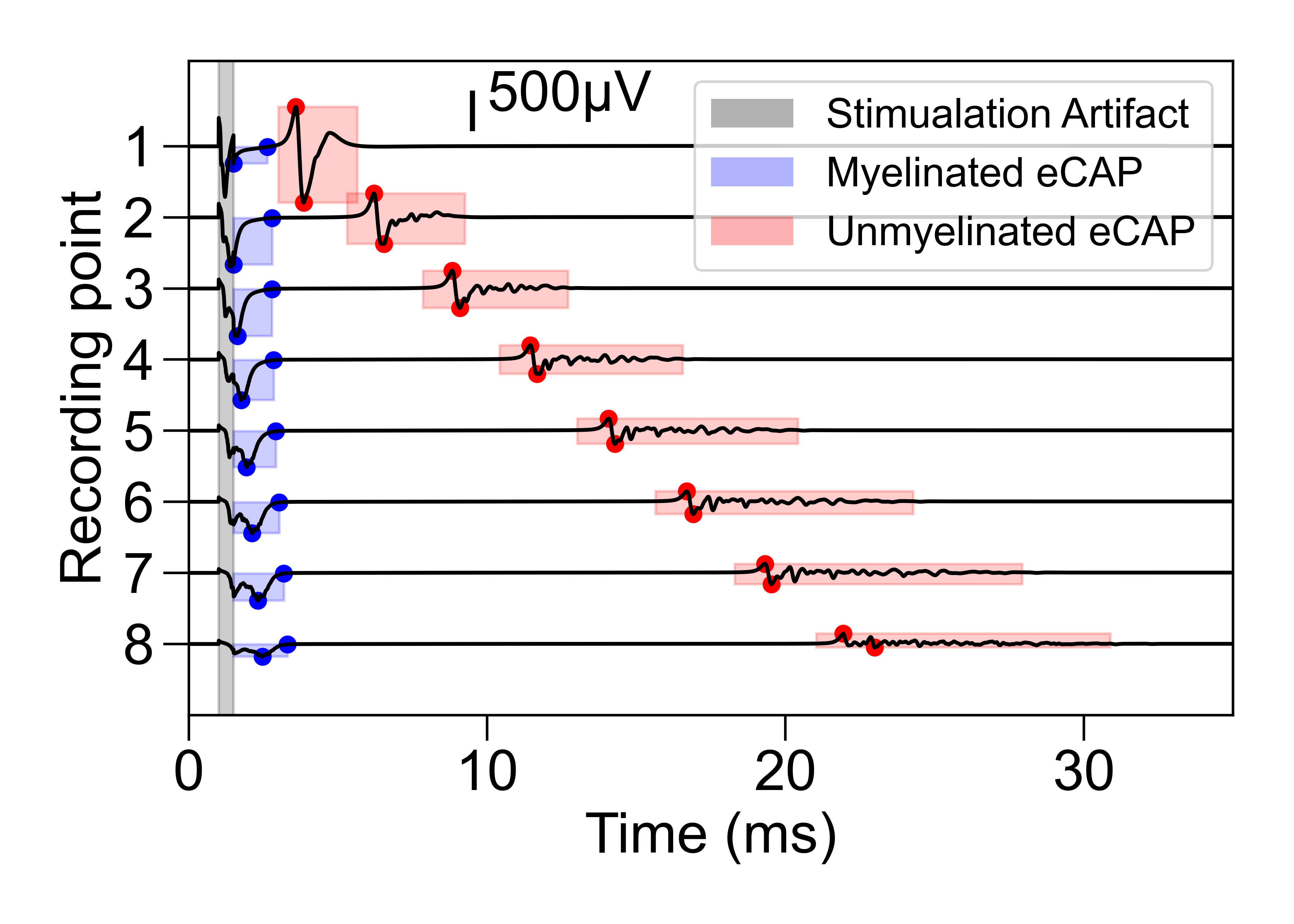

Supplement: S4 Archive — Python scripts and data files to generate and plot the in silico extracellular study. (ZIP) [file pcbi.1011826.s013.zip › S4_Archive/figures/recordings_200_4pp.png]

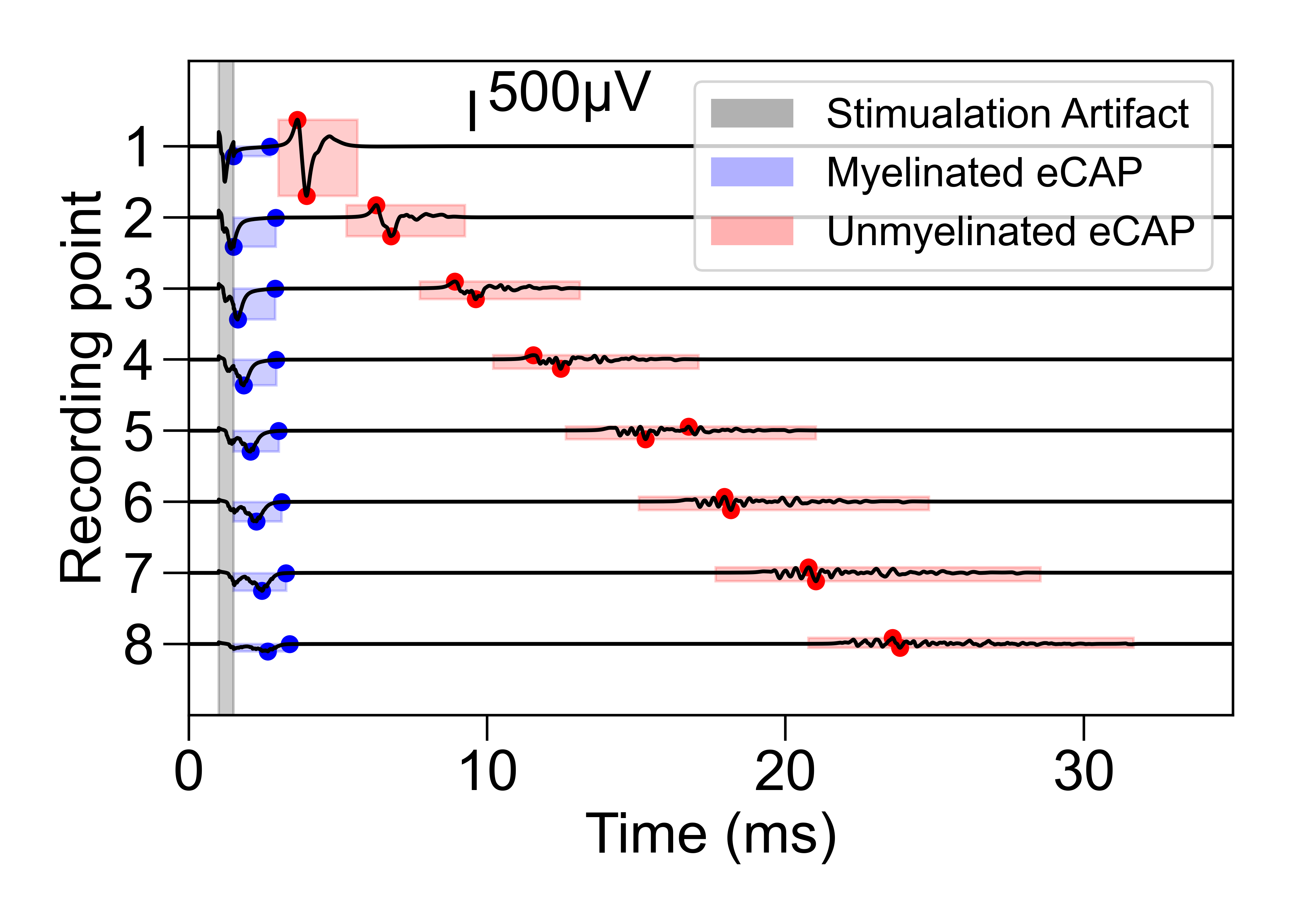

Supplement: S4 Archive — Python scripts and data files to generate and plot the in silico extracellular study. (ZIP) [file pcbi.1011826.s013.zip › S4_Archive/figures/recordings_100_2pp.png]

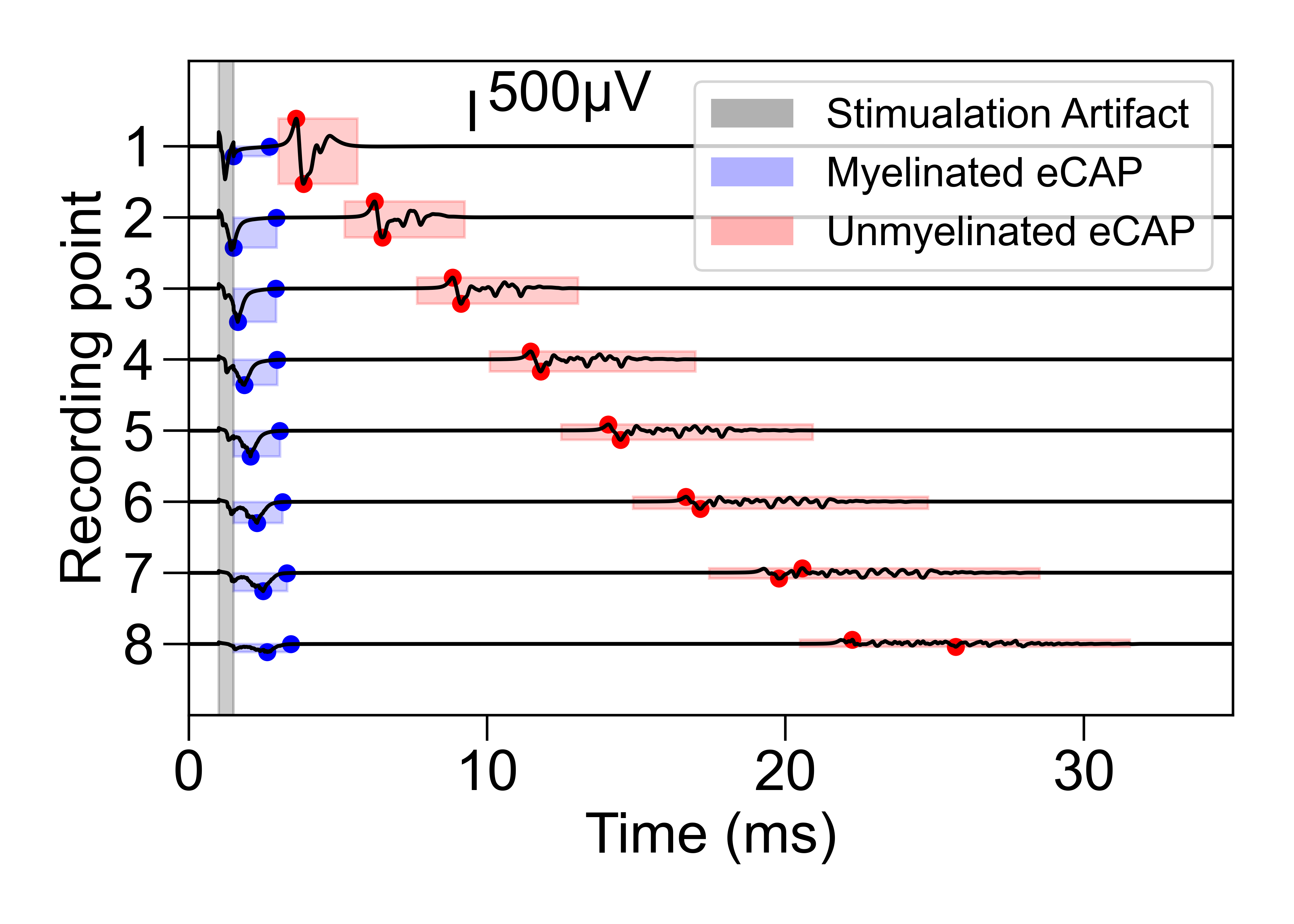

Supplement: S4 Archive — Python scripts and data files to generate and plot the in silico extracellular study. (ZIP) [file pcbi.1011826.s013.zip › S4_Archive/figures/recordings_100_4pp.png]

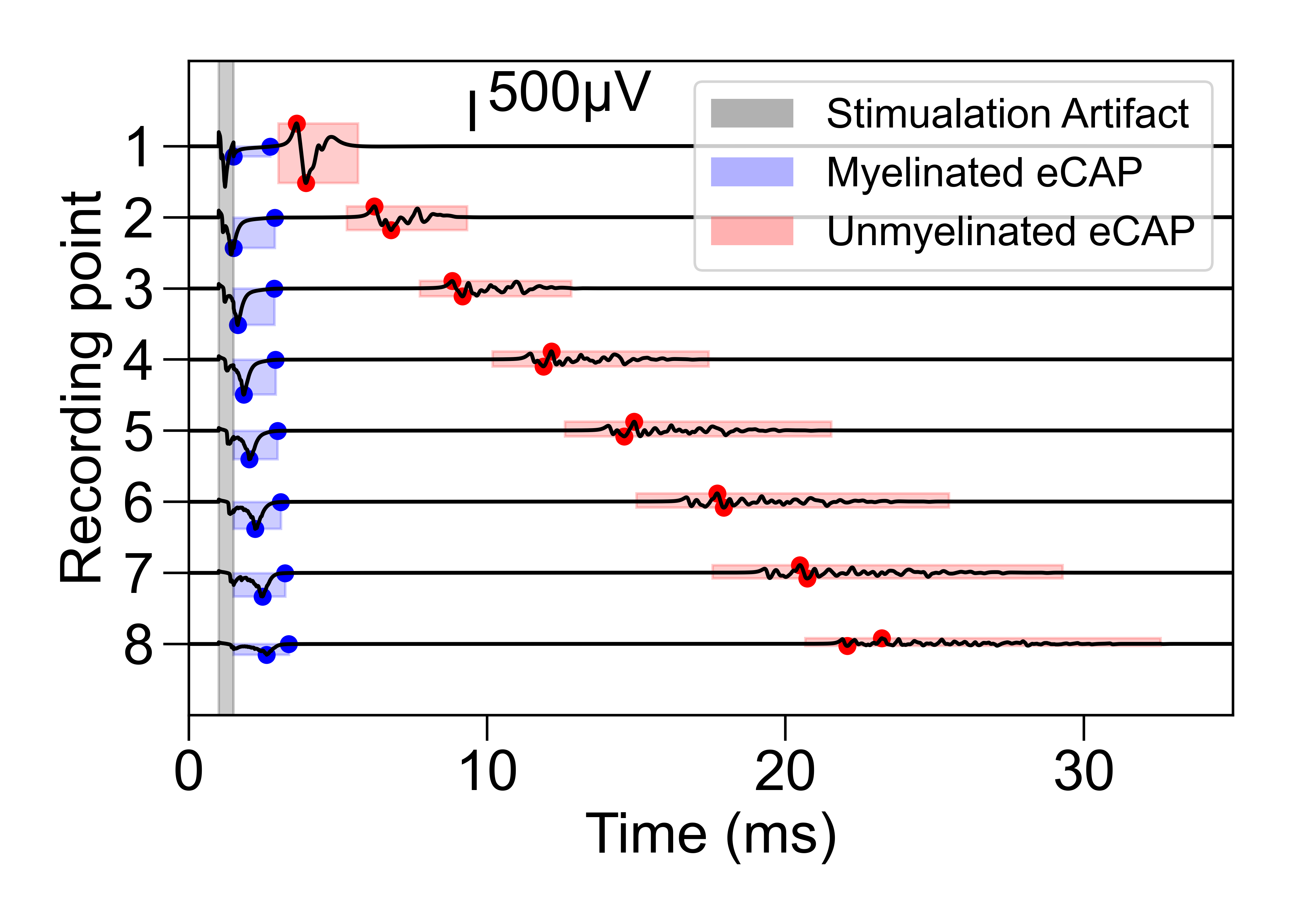

Supplement: S4 Archive — Python scripts and data files to generate and plot the in silico extracellular study. (ZIP) [file pcbi.1011826.s013.zip › S4_Archive/figures/recordings_100_3pp.png]

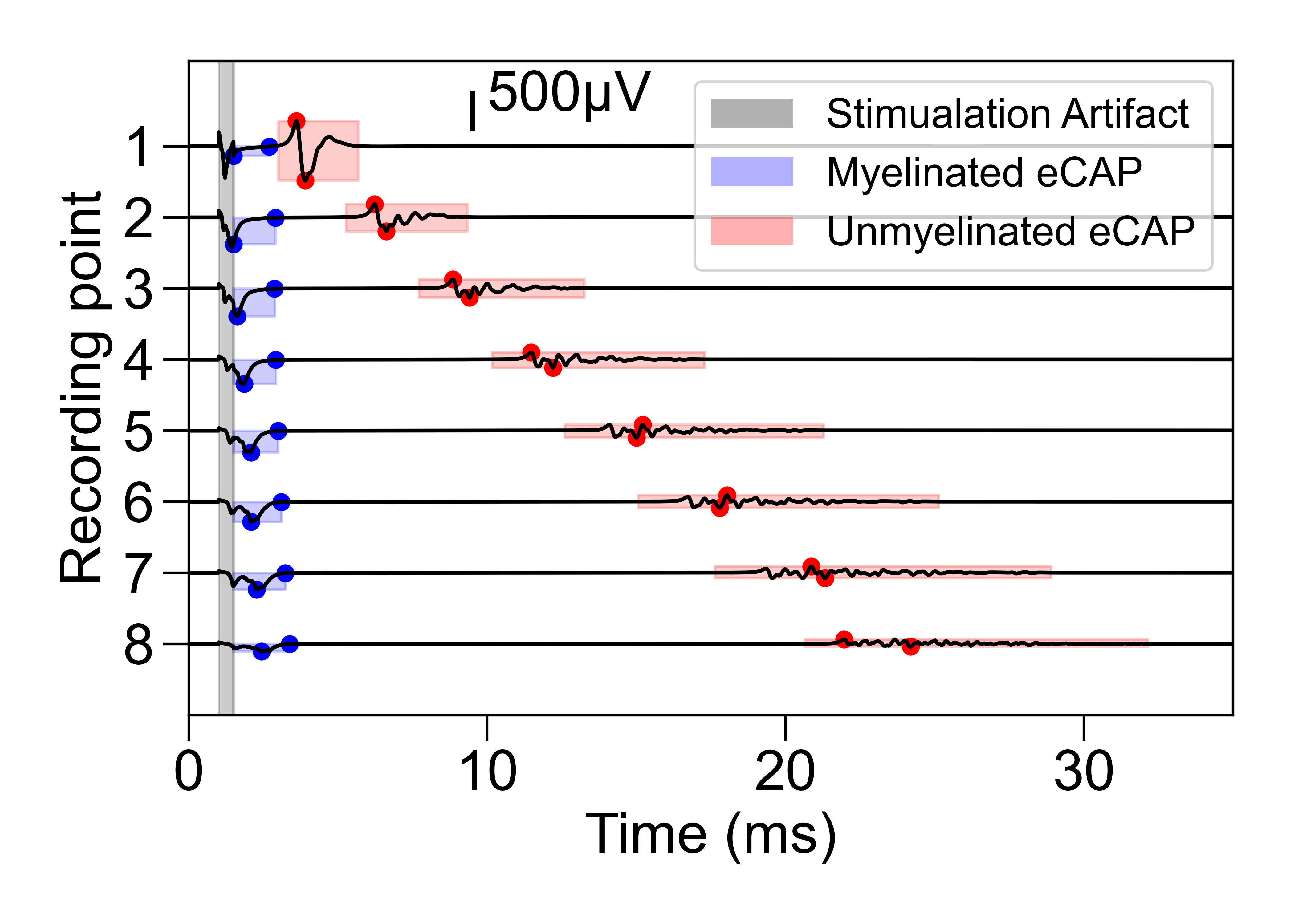

Supplement: S4 Archive — Python scripts and data files to generate and plot the in silico extracellular study. (ZIP) [file pcbi.1011826.s013.zip › S4_Archive/figures/recordings_100_1pp.png]

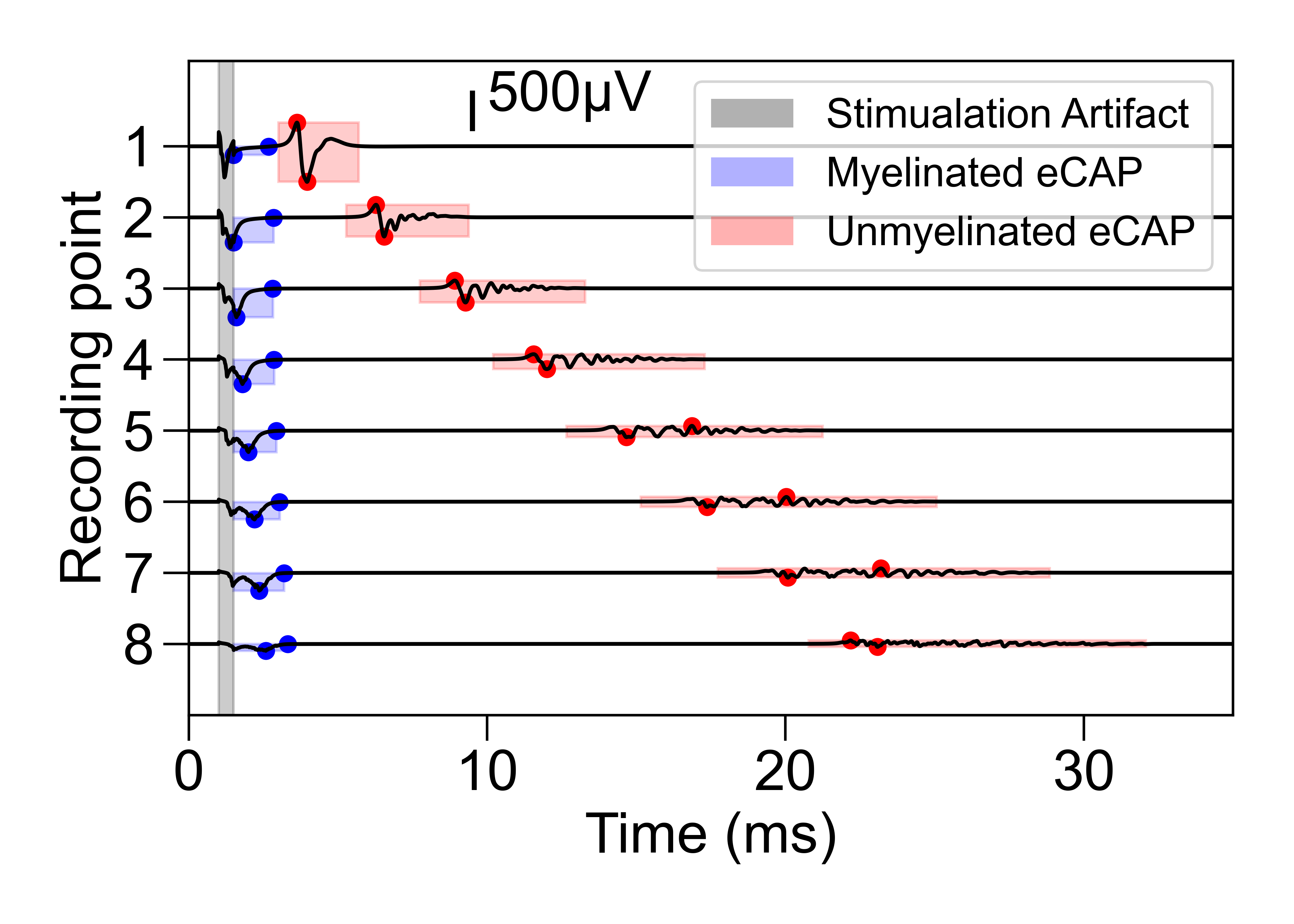

Supplement: S4 Archive — Python scripts and data files to generate and plot the in silico extracellular study. (ZIP) [file pcbi.1011826.s013.zip › S4_Archive/figures/recordings_100_5pp.png]

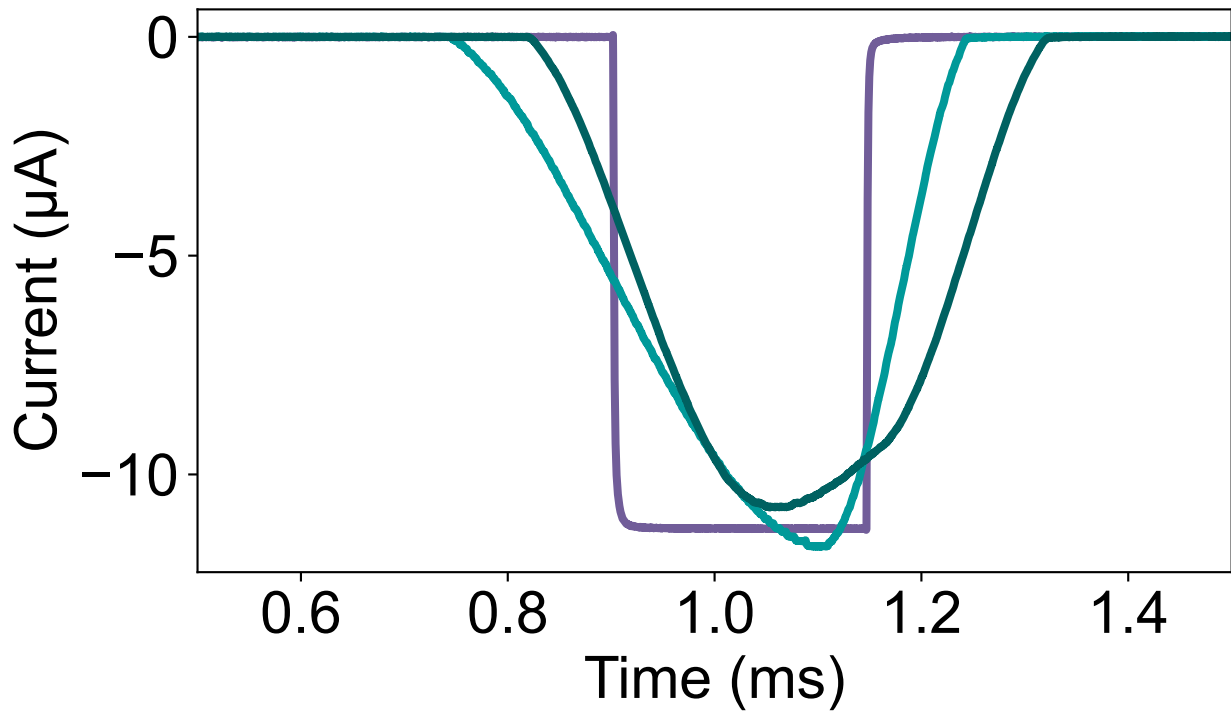

Supplement: S5 Archive — Python scripts and data files required to create and run the optimization problem, analyze the results, and translate the results to the neurostimulator. (ZIP) [file pcbi.1011826.s014.zip › S5_Archive/figures/I_recorded.pdf]

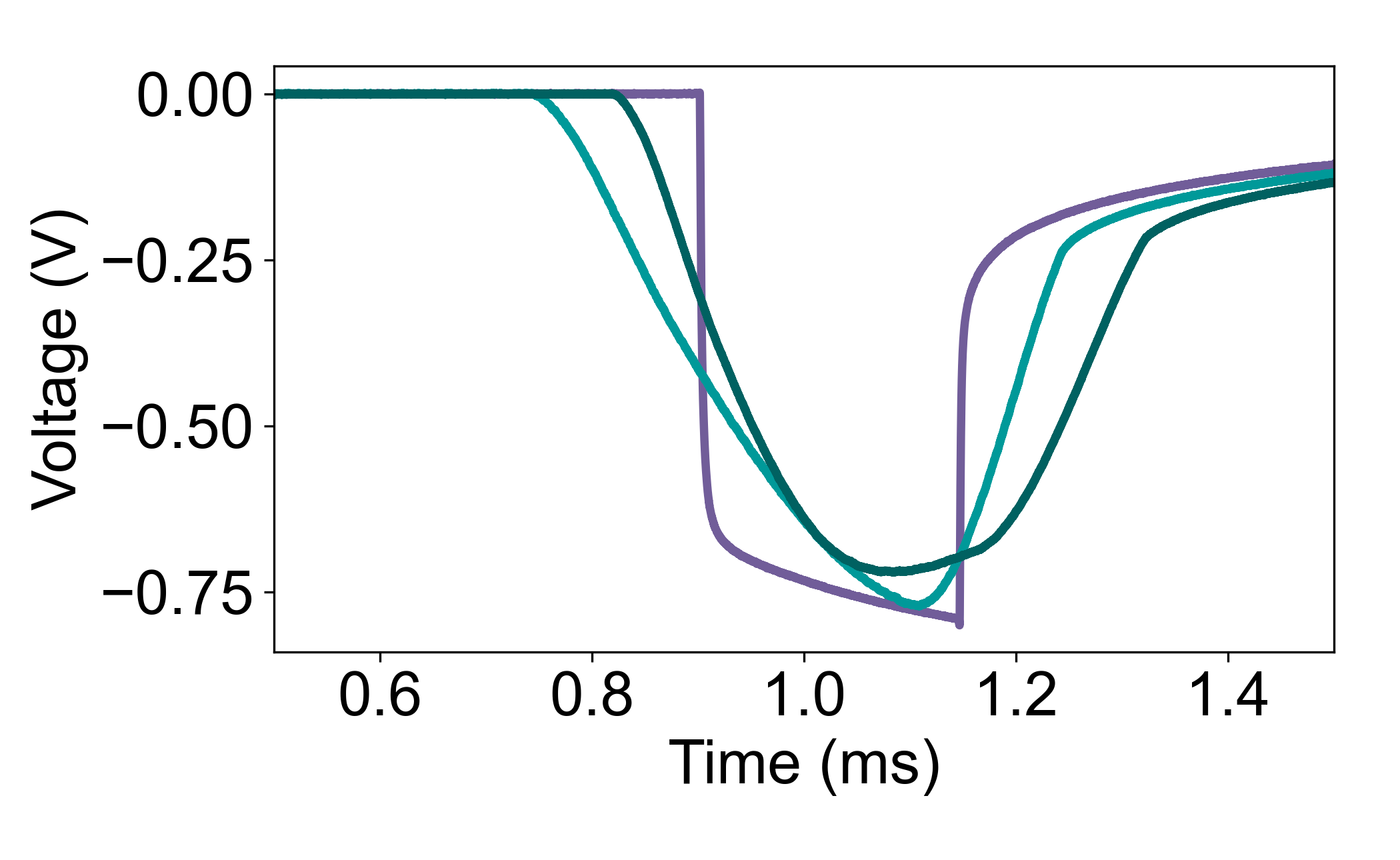

Supplement: S5 Archive — Python scripts and data files required to create and run the optimization problem, analyze the results, and translate the results to the neurostimulator. (ZIP) [file pcbi.1011826.s014.zip › S5_Archive/figures/V_recorded.png]

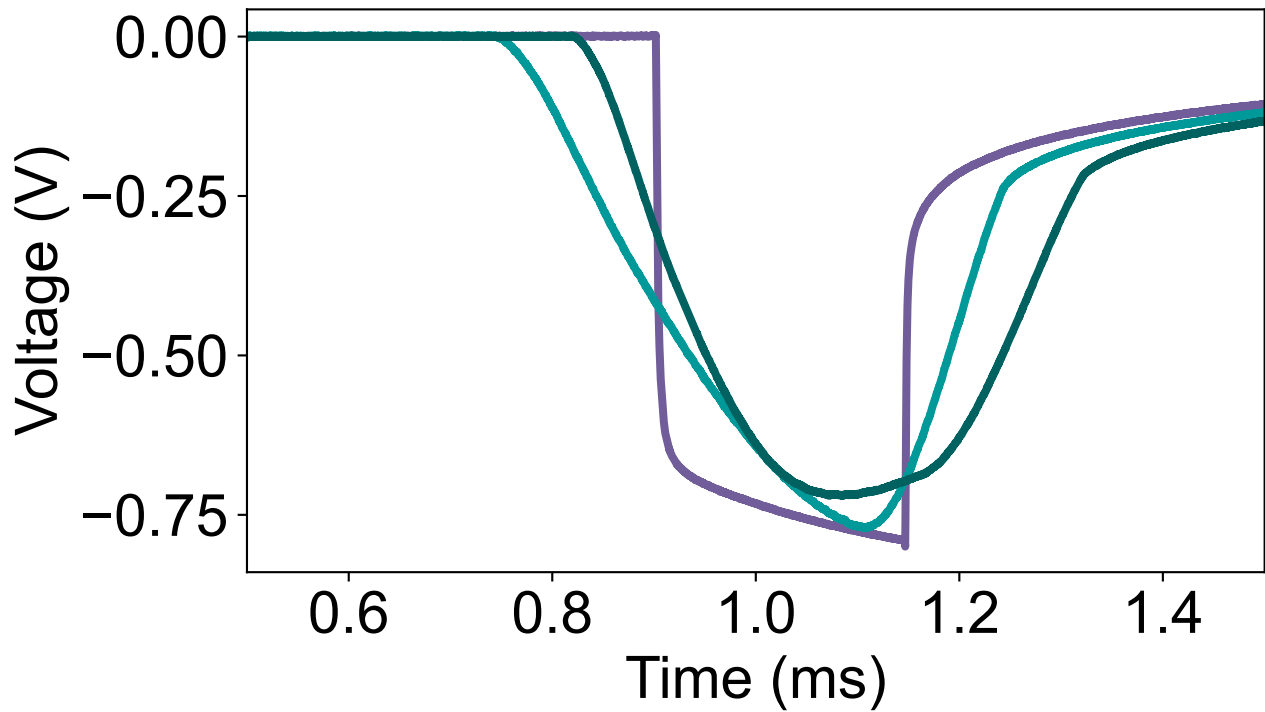

Supplement: S5 Archive — Python scripts and data files required to create and run the optimization problem, analyze the results, and translate the results to the neurostimulator. (ZIP) [file pcbi.1011826.s014.zip › S5_Archive/figures/V_recorded.pdf]

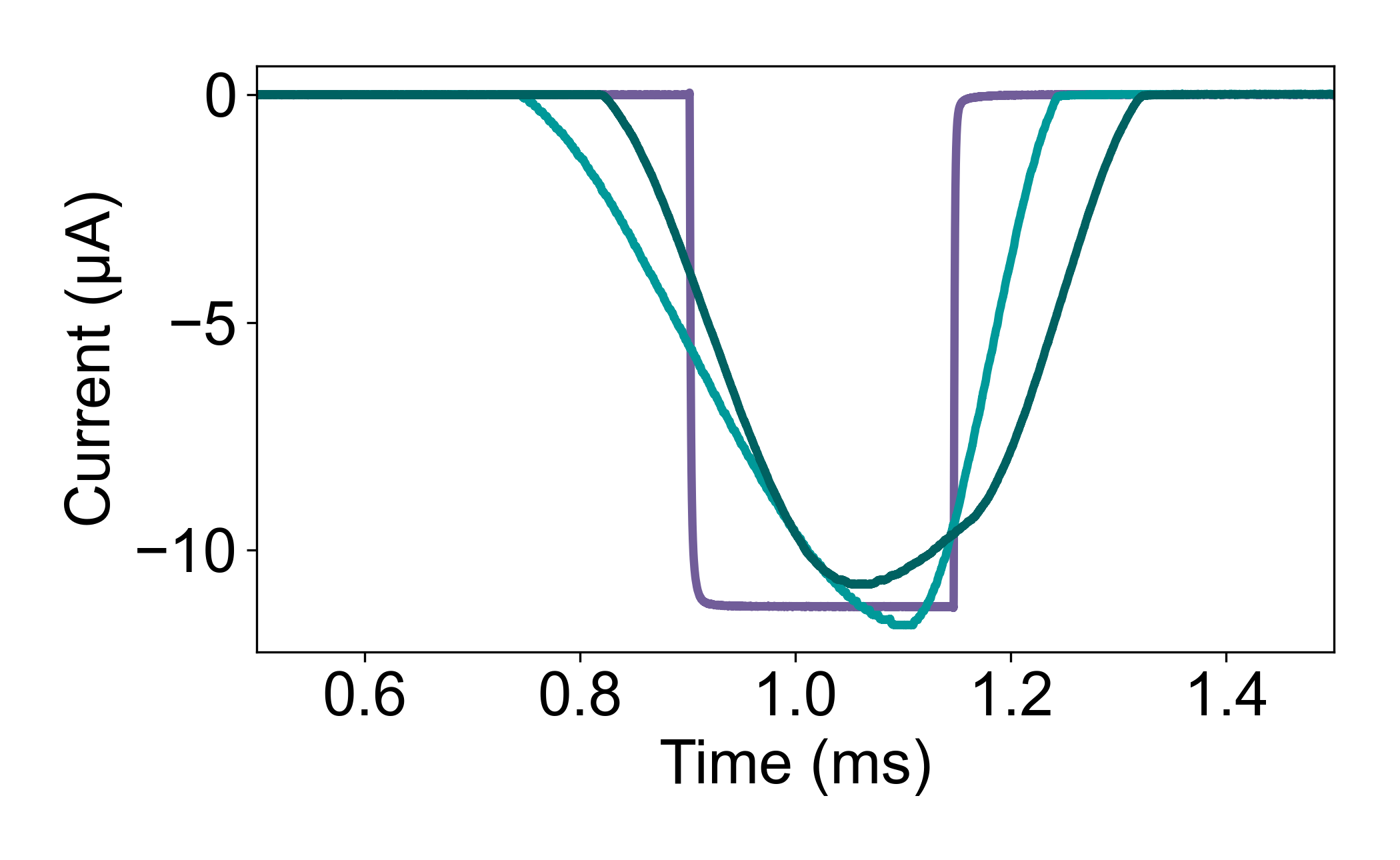

Supplement: S5 Archive — Python scripts and data files required to create and run the optimization problem, analyze the results, and translate the results to the neurostimulator. (ZIP) [file pcbi.1011826.s014.zip › S5_Archive/figures/I_recorded.png]

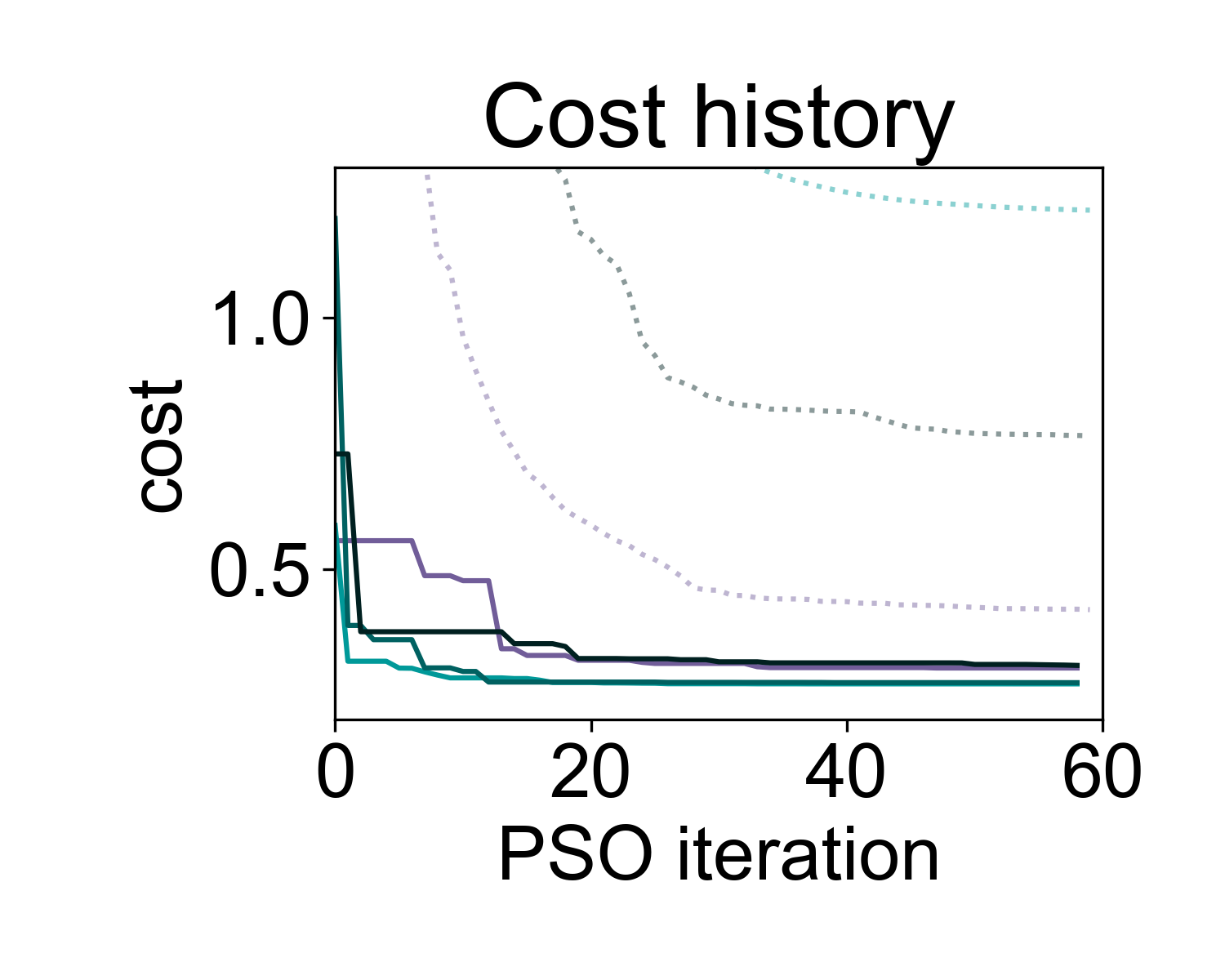

Supplement: S5 Archive — Python scripts and data files required to create and run the optimization problem, analyze the results, and translate the results to the neurostimulator. (ZIP) [file pcbi.1011826.s014.zip › S5_Archive/figures/nrj_cost_historyzoomed.png]

# Cost history

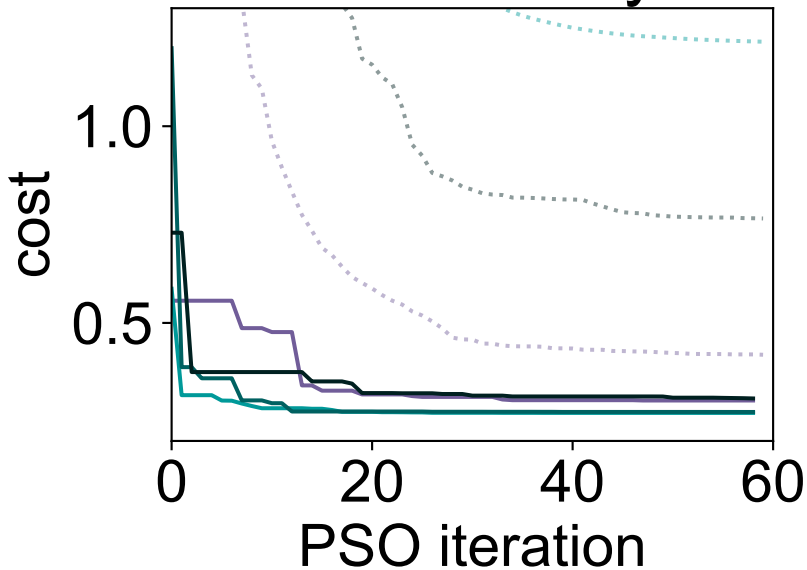

Supplement: S5 Archive — Python scripts and data files required to create and run the optimization problem, analyze the results, and translate the results to the neurostimulator. (ZIP) [file pcbi.1011826.s014.zip › S5_Archive/figures/nrj_cost_historyzoomed.pdf]

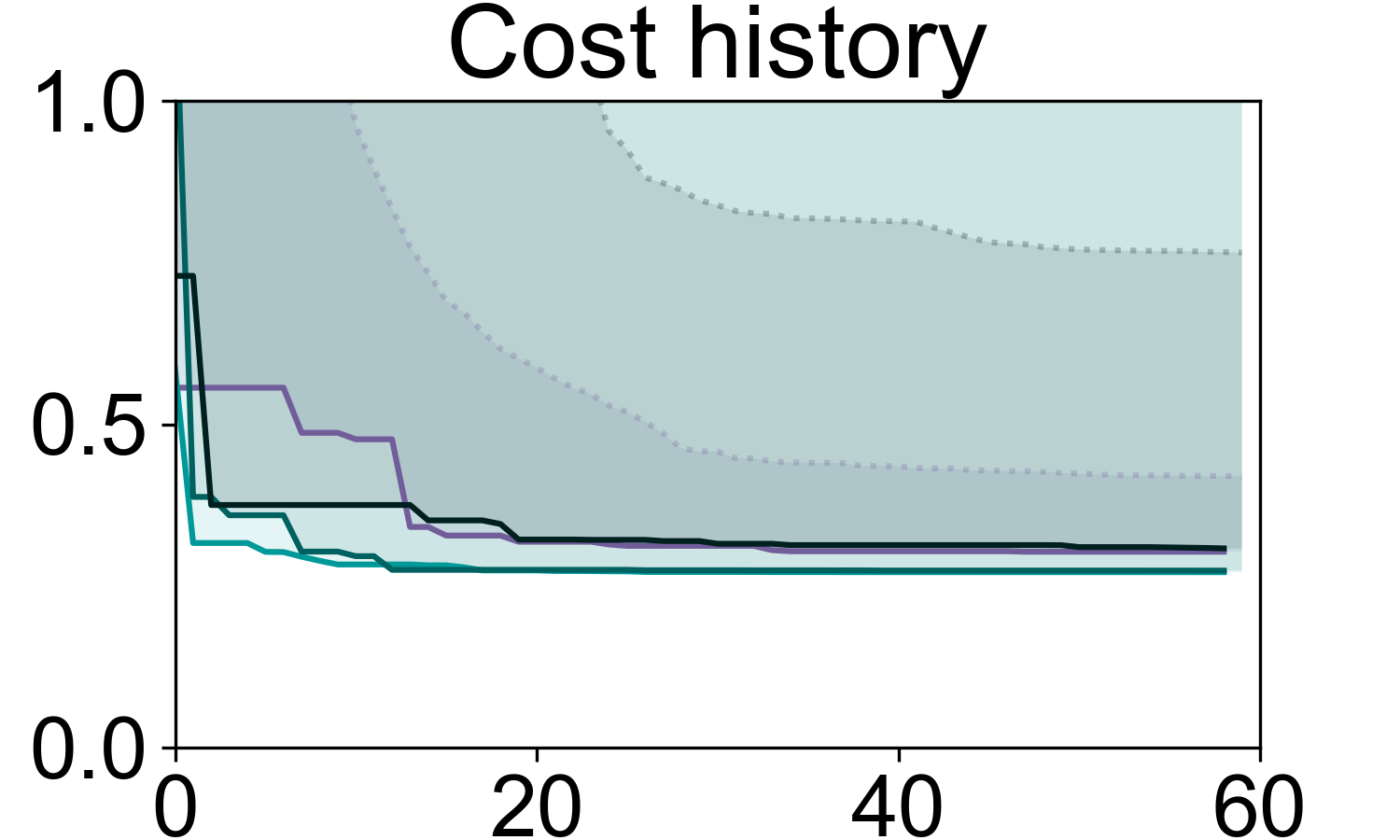

Supplement: S5 Archive — Python scripts and data files required to create and run the optimization problem, analyze the results, and translate the results to the neurostimulator. (ZIP) [file pcbi.1011826.s014.zip › S5_Archive/figures/nrj_cost_history.png]

# Cost history

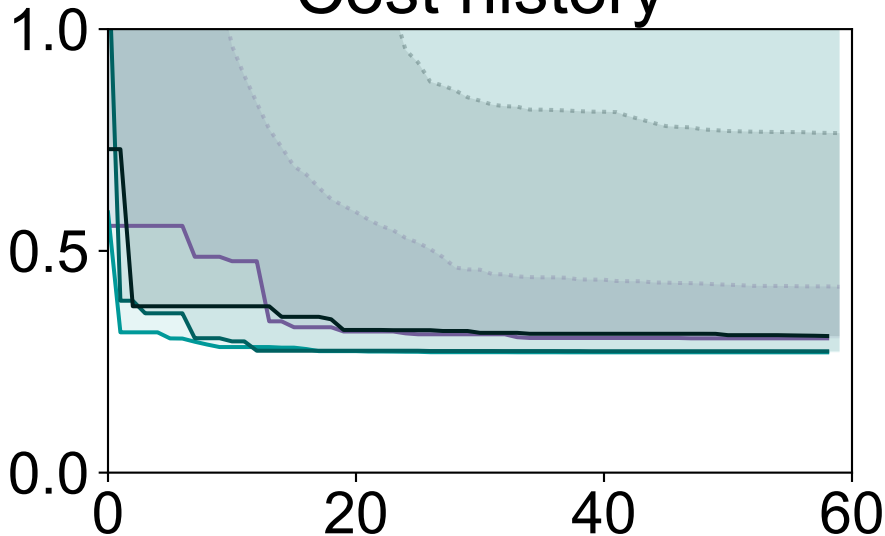

Supplement: S5 Archive — Python scripts and data files required to create and run the optimization problem, analyze the results, and translate the results to the neurostimulator. (ZIP) [file pcbi.1011826.s014.zip › S5_Archive/figures/nrj_cost_history.pdf]

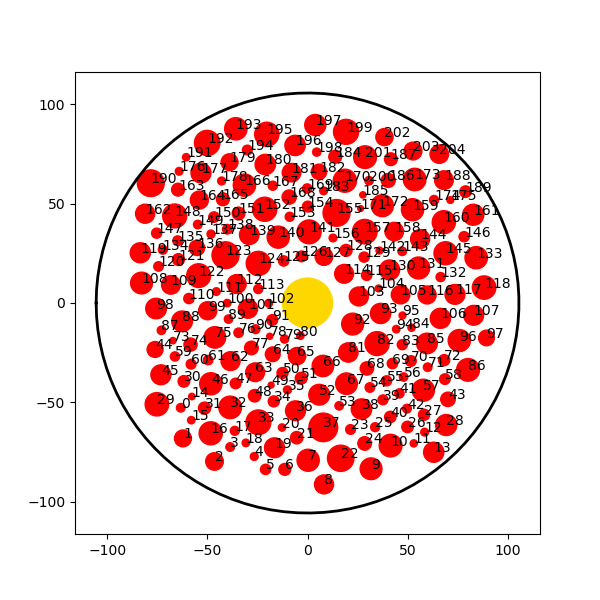

Supplement: S5 Archive — Python scripts and data files required to create and run the optimization problem, analyze the results, and translate the results to the neurostimulator. (ZIP) [file pcbi.1011826.s014.zip › S5_Archive/sources/fascicles/fascicle_M2.png]

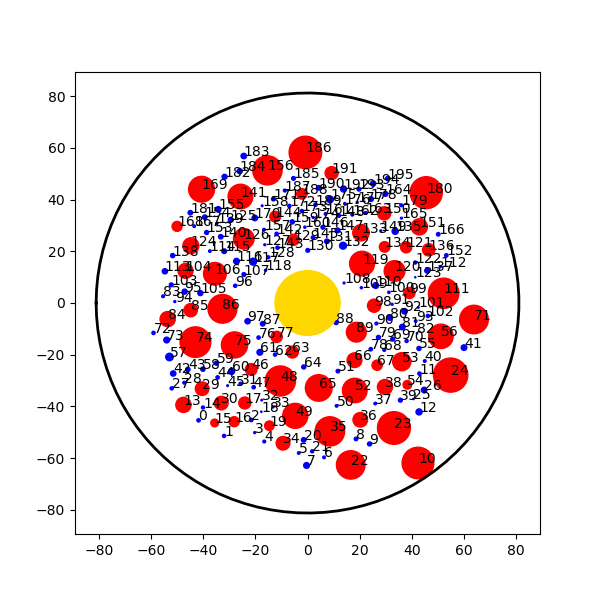

Supplement: S5 Archive — Python scripts and data files required to create and run the optimization problem, analyze the results, and translate the results to the neurostimulator. (ZIP) [file pcbi.1011826.s014.zip › S5_Archive/sources/fascicles/fascicle_1.png]
