# Supplementary material for: NRV: An open framework for in silico evaluation of peripheral nerve electrical stimulation strategies
Source: PLoS Comput Biol. 2024 Jul 12;20(7):e1011826. doi: 10.1371/journal.pcbi.1011826 (PMC11268605; doi:10.1371/journal.pcbi.1011826)
Supplement: Attachment — Submitted filename: PLOS Computational Biology Reviewer.pdf [file pcbi.1011826.s016.pdf]

The authors present a research article aiming to develop an open-source framework for in-silico study of peripheral nerve electrical stimulation using multiscale analysis. That framework could be used by the researcher and clinicians to facilitate the design of different stimulation strategies, considering the possibility of implanting multiple electrodes and testing different types of stimulation waveforms. The authors compared and validated the results obtained from their new open-source Pyhton-based framework with the results based on the most common approaches used in the literature using commercial FEM and data processing software.

The topic in consideration is relevant for the readers of PLOS Computational Biology. However, the work should be improved before being considered for acceptance in this journal, possibly taking into consideration the following issues in the revised version.

Major comments

1. In the Introduction section (i.e. lines 8-12 or line 28) or in the Results section at line 469, some important articles carried out in the same research area that used the hybrid FEM-Neuron approach are missing:
    - C. Schmidt and U. van Rienen, "Adaptive Estimation of the Neural Activation Extent in Computational Volume Conductor Models of Deep Brain Stimulation," in IEEE Transactions on Biomedical Engineering, vol. 65, no. 8, pp. 1828-1839, Aug. 2018, doi: 10.1109/TBME.2017.2758324.
    - Stefano, M., Cordella, F., Gioi, S. M. L., & Zollo, L. (2021, May). Electrical stimulation of the human median nerve: A comparison between anatomical and simplified simulation models. In 2021 10th International IEEE/EMBS Conference on Neural Engineering (NER) (pp. 769-772). IEEE.
    - Salkim, E., Shiraz, A., & Demosthenous, A. (2019). Impact of neuroanatomical variations and electrode orientation on stimulus current in a device for migraine: A computational study. Journal of neural engineering, 17(1), 016006.
    - Stefano, M., Cordella, F., Cozza, G., Ciancio, A. L., & Zollo, L. (2023). Study of electrical neural stimulation effects using extraneural and intraneural electrodes. Applied Sciences, 13(10), 6267.

2. I suggest to the authors adding the execution time needed for the different steps of a "test" simulation with fixed properties (i.e. building the geometry, meshing, FEM solution computing, evaluation of axon fibers activation).

3. At line 243, PSA is cited. Please, could you specify the conditions for which you can use the PSA?

4. At line 640, why did you use 205 myelinated axons? Please, could you motivate this choice?

Minor comments

1. At line 241, “ID” can be modified in “1D”.

2. At line 317, the authors cited the no ephaptic coupling hypothesis. Please, could you add a reference?

3. In the Section **“Perspectives”** it is reported at line 792 that “NRV only provides classes and methods to generate parameterized cylindrical nerve and fascicle geometries”. Please, could you add this information also in the section **“Our approach: NRV Overview”**?

4. In the caption of Fig. 7, could you cite the research paper that justifies the setting of the thickness of the myelin sheath?
