# Supplementary material for: NRV: An open framework for in silico evaluation of peripheral nerve electrical stimulation strategies
Source: PLoS Comput Biol. 2024 Jul 12;20(7):e1011826. doi: 10.1371/journal.pcbi.1011826 (PMC11268605; doi:10.1371/journal.pcbi.1011826)
Supplement: Attachment — Submitted filename: PLOS Comp Biol - Comment to the authors .pdf [file pcbi.1011826.s018.pdf]

Reviewer 2

The authors present a research article aiming to develop an open-source framework for in-silico study of peripheral nerve electrical stimulation using multiscale analysis. That framework could be used by the researcher and clinicians to facilitate the design of different stimulation strategies, considering the possibility of implanting multiple electrodes and testing different types of stimulation waveforms. The authors compared and validated the results obtained from their new open-source Python-based framework with the results based on the most common approaches used in the literature using commercial FEM and data processing software. The topic in consideration is relevant for the readers of PLOS Computational Biology. However, the work should be improved before being considered for acceptance in this journal, possibly taking into consideration the following issues in the revised version.

Authors: We would like to thank the reviewers for their careful review. We have addressed all of the comments and detail how we have changed the text below.

R: Note that in the second draft the line numbers in the firsts two pages are missing, so all the other line numbers are shifted respect to the comments of the authors. Because of the information in the comments and to the different text color in the draft it is still possible to check the author responses.

Major comments

1. In the Introduction section (i.e. lines 8-12 or line 28) or in the Results section at line 469, some important articles carried out in the same research area that used the hybrid FEM-Neuron approach are missing:

   - C. Schmidt and U. van Rienen, "Adaptive Estimation of the Neural Activation Extent in Computational Volume Conductor Models of Deep Brain Stimulation," in IEEE Transactions on Biomedical Engineering, vol. 65, no. 8, pp. 1828-1839, Aug. 2018, doi: 10.1109/TBME.2017.2758324.
   - Stefano, M., Cordella, F., Gioi, S. M. L., & Zollo, L. (2021, May). Electrical stimulation of the human median nerve: A comparison between anatomical and simplified simulation models. In 2021 10th International IEEE/EMBS Conference on Neural Engineering (NER) (pp. 769-772). IEEE.
   - Salkim, E., Shiraz, A., & Demosthenous, A. (2019). Impact of neuroanatomical variations and electrode orientation on stimulus current in a device for migraine: A computational study. Journal of neural engineering, 17(1), 016006.
   - Stefano, M., Cordella, F., Cozza, G., Ciancio, A. L., & Zollo, L. (2023). Study of electrical neural stimulation effects using extraneural and intraneural electrodes. Applied Sciences, 13(10), 6267.

Authors: These references have been added to the text and correspond to the numbers 35, 36, 37, and 38, respectively (line 31).

R1: The authors satisfied the response.

2. I suggest to the authors adding the execution time needed for the different steps of a "test" simulation with fixed properties (i.e. building the geometry, meshing, FEM solution computing, evaluation of axon fibers activation).

Authors: Thanks for the insightful comment. We have written a benchmark script and provided timing results on various machines, from laptops to clusters. This corresponds to Table 2 and is detailed in the discussion section (lines 788 to 791).

R2: I am satisfied with the response.

3. At line 243, PSA is cited. Please, could you specify the conditions for which you can use the PSA?

Authors: We mentioned in the text the limitations and a citation (reference number 61, line 259).

R3: I am satisfied with the response.

4. At line 640, why did you use 205 myelinated axons? Please, could you motivate this choice?

Authors: We have described in the text the process used to generate the neural population for the optimization result (lines 645 to 649). It corresponds to the number needed to pack a 200µm in diameter fascicle. This number is arbitrary, but large enough to demonstrate the optimization capabilities of NRV on a non-trivial problem.

R4: I am satisfied with the response.

Minor comments

1. At line 241, "ID" can be modified in "1D".

Authors: ID is the correct terminology. We identify each electrode of the model with a unique number so multiple electrodes can be used with individual stimulus. We clarified in the text, changing "ID" with "identifier" (line 254).
R1: I am satisfied with the comment. Thank you for the response.

2. At line 317, the authors cited the no ephaptic coupling hypothesis. Please, could you add a reference?

Authors: We have added reference numbers 34, 46, and 55 to justify this hypothesis in the introduction of the document (line 41).
R2: I am satisfied with the response.

3. In the Section **“Perspectives”** it is reported at line 792 that “NRV only provides classes and methods to generate parameterized cylindrical nerve and fascicle geometries”. Please, could you add this information also in the section **“Our approach: NRV Overview”**?

Authors: We have added this information in the appropriate paragraph (line 166) to avoid any misleading information. Thank you.

R3: I am satisfied with the response.

4. In the caption of Fig. 7, could you cite the research paper that justifies the setting of the thickness of the myelin sheath?

Authors: We assume you meant perineurium, not myelin sheath? If not, then the myelin sheath thickness is defined by the MRG model (cited earlier in the paper). While checking this, we also noticed that the chosen perineurium thickness was too low; we changed this value according to the literature (reference 18, perineurium to 3% of fascicle diameter = 24µm) and updated the full figure and results with the corresponding simulations.

R4: Thank you for the response, there was a mismatch between myelin sheath and perineurium. I referred to the thickness of the perineurium because it looked too low. Now, it is in agree with the literature. I am satisfied with the response.
