# Supplementary material for: NRV: An open framework for in silico evaluation of peripheral nerve electrical stimulation strategies
Source: PLoS Comput Biol. 2024 Jul 12;20(7):e1011826. doi: 10.1371/journal.pcbi.1011826 (PMC11268605; doi:10.1371/journal.pcbi.1011826)
Supplement: Attachment — Submitted filename: nrv.docx [file pcbi.1011826.s015.docx]

Overall, the manuscript is far from being ready for publication and this reality precluded a thorough (and timely) review. The manuscript is littered with typos, grammar errors, inaccurate/incomplete/misleading statements on prior art, and redundancies. The descriptions of software implementation and intended use are incomplete, and the documentation is not complete enough to understand NRV implementation and user control of simulations. Key citations are missing.

Some notes on from my first pass of review, which are not exhaustive for a thorough review.

- Introduction
  - REF 1 (Reardon 2014) is out of date, please cite more recent literature in addition to this article.
  - Several unnecessary capitalizations (e.g., Central Nervous System, Deep Brain Stimulation, Peripheral Nervous System)
  - “Many of them fail” but only cite one article of VNS for HF
  - “Sizeable morphological variety across species and within individuals” cite <https://pubmed.ncbi.nlm.nih.gov/33250710/>, <https://pubmed.ncbi.nlm.nih.gov/37257454/>
  - This sentence is an incomplete thought. Why does electrode design/placement and electrical stimulus matter? What do you mean by predictability?
    - “*Also, the electrode design and its spatial location and orientation, as well as the choice of the electrical stimulus will further increase the lack of predictability of the therapy.”*
  - Another (of many) REFs that would be valuable to add for context: <https://pubmed.ncbi.nlm.nih.gov/36649655/>. This sentence suggests performing in vivo experiments is not ethical.
    - “Computational modeling has proven to be a valuable tool to tackle those issues but also for accelerating the design of the therapy while containing the financial and ethical cost of the in-vivo experiments [10–13].”
  - “in-silico” and “in-vivo” do not require the “-”.
  - Strange diction with “consensual”:
    - “The process for simulating the PNS response to electrical stimuli is now rather consensual and is based on a 2-step hybrid model”
  - “It includes the anatomical and physiological features as well as an accurate representation of the electrode design and location in or around the neural tissue.”
    - 3D model does not usually include physiological features?
  - Disagree with this statement:
    - “Moreover, there is currently no framework that is universally used by the research community, reducing the possibility of experiment replications and the reuse of data [24].”
      - Options exists and are mentioned later in the paper (PyPNS, ASCENT, TxBDC, ViNERS, Sim4Life). What are they missing that NRV delivers? State that here.
  - Strongly disagree with this statement:
    - “Multi-software and/or multi-programming language approach also makes the deployment of hybrid modeling frameworks complex on the super-computer cluster or massively parallel machines.”
  - Unclear what is meant by this sentence:
    - “It can be used with a few lines of code (in Python language) and used on various computing supports, from conventional machines to large supercomputers without impacting the simulation description.”
  - Typo:
    - “describes the architecture of the our framework”
- Methods
  - Disagree with the following statement:
    - “However, no out-of-the-box framework is provided making the deployment of workflow challenging”
  - The descriptions of the various softwares are incomplete and cover inconsistent features. A reader unfamiliar with these resources may think one software does not have features because they are not mentioned, but they are mentioned for another software.
    - Just one example of many: TxBDC “can run simple parametrized simulations to estimate electrical stimulation’s recruitment curves”, but this can be done with all of these softwares mentioned (PyPNS, ASCENT, ViNERS, Sim4Life)
  - ASCENT: edit to “requires a commercial license for COMSOL”
  - TxBDC: note that Gmsh and FEniCS are open source and do not require a license
  - ViNERS:
    - Add ASCENT here: “Similarly to PyPNS and ASCENT, ViNERS can simulate electrical stimulation and neural activity recordings.”
      - ASCENT v1.3.0+ can simulate recording from cuff electrodes, as demonstrated in <https://journals.plos.org/ploscompbiol/article?id=10.1371/journal.pcbi.1011833>
    - Note that Gmsh and Eidors are open source and do not require a license
    - Not clear what you mean by “complete”:
      - “ViNERS is, to the best of our knowledge, the most complete open-source framework available, as all the required steps can be done within the toolbox.”
  - Page 5, line 151: “ASCEND” should be “ASCENT”
  - Our approach: NRV Overview
    - What do you mean by “capability of performing all the required steps for hybrid modeling within the framework”?
      - From my understanding , ViNERS and ASCENT accomplish the goal. If there is something they lack in the framework that NRV delivers, this was not made clear in the introduction.
    - “Large-scale nerve simulations are embarrassingly parallel problems and can greatly reduce computation time. NRV provides multiprocessing capabilities by implementing a message-passing interface (MPI) for Python [35]. Parallel computing is performed independently from the simulation description and the end-user only needs to provide the maximum number of usable cores on the machine; NRV automatically handles job distribution and synchronicity between the processes.”
      - Why is MPI needed for an embarrassingly parallel problem? What information is being passed between processes? Unclear what the impact is, and what is novel compared to existing solutions.
    - “Third-party libraries used are fully encapsulated in the NRV framework”
      - What does this mean? And how does this make these softwares more transparent? What is “transparency”?
  - Fmod
    - “The stimulation current Istim,k is described by a dedicated stimulus-class that defines asynchronous stimulus current and time”
      - Current is a function of time, and therefore the word asynchronous is not correct.
    - Equation 1: confusing notation because V1=I*V2, where V2 (i.e., footprint) is actually V/Amp.
      - I’ve seen this written with “transfer resistance” (Rtrans) where unitary current is delivered, and the resistance between current source and point is calculated using the voltage sensed. Then Vext = Iapplied * Rtrans. From my experience in teaching, this notation is more accessible to people new to the field.
    - Mechanics of implementation not clear:
      - “Arithmetic operations between Stimulus-objects are defined for the class, thus enabling fully customized arbitrary stimulating waveforms”
    - Figure 2 caption: word missing?
      - “NRV’s transcription of the physical with dedicated classes that can be combined and are used by the framework to evaluate the extracellular electrical fields”
    - Typo Page 9 “perineum” should be “perineurium”
    - To my knowledge, conductivities for epineurium, endoneurium, and perineurium are not all found in [40].
      - What are the actual values used for conductivity?
    - However, it restricts the geometry to only one material and the electrode shape is not taken into account, limiting the viability of this approach to model complex experimental or therapeutic setups [43].
      - Also nerve geometry and electrode interface are not taken into account
    - I do not see a reason for not including the FEM computed potentials to simulate recording? Why limit to analytical? I do not think this is a step-forward as tools for modeling recording from analytical/FEM solution already exist
    - In NRV, morphological values in [21] and from [51] are interpolated with cubic or quadratic functions
      - Specifically which values? What are the relationships used?
    - What are the statistics you used?
      - “Statistics taken from [55–57] have been interpolated and predefined as population-generating functions.”
- Optimize
  - Unclear how user actually interfaces with these features
- Results
  - Can’t find contents of Appendix 1, S2 Files
  - What are red dots in Figure 7a and 7b?
  - Citation instruction to converge for thresholds, not voltage or its 2^nd^ difference: <https://pubmed.ncbi.nlm.nih.gov/24448594/>
- Scalability:
  - But NRV *does* depend on external software… e.g., NEURON, Python, optimization packages, etc…
  - You said the NEURON simulations are embarrassingly parallel, so how do you speed up the simulations?

Overall

- Documentation for installation and use is not clear or thorough. What do I change to customize the follow (as example, not exhaustive list):
  - Waveform
  - Material property
  - Fiber model
  - Cuff
  - Cost function
  - Etc
- Main text is difficult to follow, simultaneously not enough detail to understand how it works (need documentation for users) but also not streamlined enough to appreciate what has been built.
