# Supplementary material for: NRV: An open framework for in silico evaluation of peripheral nerve electrical stimulation strategies
Source: PLoS Comput Biol. 2024 Jul 12;20(7):e1011826. doi: 10.1371/journal.pcbi.1011826 (PMC11268605; doi:10.1371/journal.pcbi.1011826)
Supplement: Attachment — Submitted filename: letter_to_reviewers.docx [file pcbi.1011826.s017.docx]

Dear Editor and Reviewers,

We would like to thank you and the reviewers for the valuable comments concerning our manuscript “PCOMPBIOL-D-24-00070”. We have carefully considerate every comment and have made correction accordingly. We sincerely hope that those corrections will meet your standards. Any further constructive comments are welcome. Revised portion of the manuscript are marked in red. Please find below point-by-point responses to the reviewer’s comments.

# Reviewer 1

**Reviewer 1:** Overall, the manuscript is far from being ready for publication and this reality precluded a thorough (and timely) review. The manuscript is littered with typos, grammar errors, inaccurate/incomplete/misleading statements on prior art, and redundancies. The descriptions of software implementation and intended use are incomplete, and the documentation is not complete enough to understand NRV implementation and user control of simulations. Key citations are missing.

**Authors:** We thank Reviewer 1 for his very careful review. We have attempted to address each of the suggested points, and our changes/answers are detailed below, with links to the current line numbers involved.

## Introduction

**Reviewer 1:** Some notes on from my first pass of review, which are not exhaustive for a thorough review. REF 1 (Reardon 2014) is out of date, please cite more recent literature in addition to this article.

**Authors:** We included 4 additional references (1 to 5, line 5).

**Reviewer 1:** Several unnecessary capitalizations (e.g., Central Nervous System, Deep Brain Stimulation, Peripheral Nervous System)

**Authors:** Thank you. We removed unnecessary capitalization.

**Reviewer 1:** “Many of them fail” but only cite one article of VNS for HF

**Authors:** Added additional literature references, cited papers now include references 12 through 17 (line 17).

**Reviewer 1:** “Sizeable morphological variety across species and within individuals” cite <https://pubmed.ncbi.nlm.nih.gov/33250710/>, <https://pubmed.ncbi.nlm.nih.gov/37257454/>

**Authors:** These suggested papers have been added to the bibliography (ref number 19-20, line 19).

**Reviewer 1:** This sentence is an incomplete thought. Why does electrode design/placement and electrical stimulus matter? What do you mean by predictability?

“*Also, the electrode design and its spatial location and orientation, as well as the choice of the electrical stimulus will further increase the lack of predictability of the therapy.”*

**Authors:** We added references 21 (electrode placement), 22 (electrode position), 23 (stimulus shape) to justify these elements (line 21).

**Reviewer 1:** Another (of many) REFs that would be valuable to add for context: <https://pubmed.ncbi.nlm.nih.gov/36649655/>.

**Authors:** We have added this reference as a use case example for computational models (reference number 28, line 23). Please note that we do not intend to review all use cases for in silico modeling, but only to justify the need for in silico models in the introduction.

**Reviewer 1:** This sentence suggests performing in vivo experiments is not ethical.

“Computational modeling has proven to be a valuable tool to tackle those issues but also for accelerating the design of the therapy while containing the financial and ethical cost of the in-vivo experiments [10–13].”

**Authors:** We have rephrased the sentence to clarify our statement (lines 23 to 26). A reference to the four-R rules have been added.

**Reviewer 1:** “in-silico” and “in-vivo” do not require the “-”.

**Authors:** We have removed the "-" from the entire document and hope we have not missed any occurrences.

**Reviewer 1:** Strange diction with “consensual”:

“The process for simulating the PNS response to electrical stimuli is now rather consensual and is based on a 2-step hybrid model”

**Authors:** This sentence has been rephrased (lines 30 to 31). Thank you for pointing out this poor wording.

**Reviewer 1:** “It includes the anatomical and physiological features as well as an accurate representation of the electrode design and location in or around the neural tissue.”

- 3D model does not usually include physiological features?

**Authors:** We have removed the word physiological (line 32), as we understand that it can be confused with physiological features of the axon membrane (not included in the FEM model). Thank you for highlighting this.

**Reviewer 1:** Disagree with this statement:

“Moreover, there is currently no framework that is universally used by the research community, reducing the possibility of experiment replications and the reuse of data [24].”

Options exists and are mentioned later in the paper (PyPNS, ASCENT, TxBDC, ViNERS, Sim4Life). What are they missing that NRV delivers? State that here.

Strongly disagree with this statement:

“Multi-software and/or multi-programming language approach also makes the deployment of hybrid modeling frameworks complex on the super-computer cluster or massively parallel machines.”

**Authors:** We have rewritten this entire section and moved it to the end of “Overview of existing solutions” (lines 141 to 156). We have emphasized the fact that ready-to-use solutions are now available, but rely on commercial solutions. Fully open-source solutions are also possible, but require gathering all available but spare solutions (e.g. gmsh, fenics, neuron) before running the simulation. We hope this statement is now clearer.

**Reviewer 1:** Unclear what is meant by this sentence:

“It can be used with a few lines of code (in Python language) and used on various computing supports, from conventional machines to large supercomputers without impacting the simulation description.”

**Authors:** We rewrote the sentence (lines 46 to 51). We hoped to be more specific.

**Reviewer 1:** Typo

“describes the architecture of the our framework”

**Authors:** It is corrected, thank you.

## Methods

**Reviewer 1:** Disagree with the following statement:

“However, no out-of-the-box framework is provided making the deployment of workflow challenging”

**Authors:** We rephrased and emphasized that although Raspopovic et al. and Romeni et al. provided clear guidelines for modeling, they did not provide practical solutions (lines 70 to 71). We also state that out of the box solutions are now available, with limitations that we introduce at the end of this section (lines 141 to 156).

**Reviewer 1:** The descriptions of the various softwares are incomplete and cover inconsistent features. A reader unfamiliar with these resources may think one software does not have features because they are not mentioned, but they are mentioned for another software.

Just one example of many: TxBDC “can run simple parametrized simulations to estimate electrical stimulation’s recruitment curves”, but this can be done with all of these softwares mentioned (PyPNS, ASCENT, ViNERS, Sim4Life)

ASCENT: edit to “requires a commercial license for COMSOL”

TxBDC: note that Gmsh and FEniCS are open source and do not require a license

Not clear what you mean by “complete”: “ViNERS is, to the best of our knowledge, the most complete open-source framework available, as all the required steps can be done within the toolbox.”

**Authors:** We thank the reviewer for these comments about reviewing existing frameworks and pipelines. We have completely rewritten this section with these comments in mind (lines 75 to 156)

**Reviewer 1:** ViNERS: Add ASCENT here: “Similarly to PyPNS and ASCENT, ViNERS can simulate electrical stimulation and neural activity recordings.”

ASCENT v1.3.0+ can simulate recording from cuff electrodes, as demonstrated in <https://journals.plos.org/ploscompbiol/article?id=10.1371/journal.pcbi.1011833>

**Authors:** We include this reference (number 51 in the text), however we want to point out this article was published on March the 1^st^, and our submission was on January the 12th ; we could not have this information for initial submission.

**Reviewer 1:** Page 5, line 151: “ASCEND” should be “ASCENT”

**Authors:** We corrected this typo, thank you for this remark.

**Reviewer 1:**

What do you mean by “capability of performing all the required steps for hybrid modeling within the framework”? From my understanding , ViNERS and ASCENT accomplish the goal. If there is something they lack in the framework that NRV delivers, this was not made clear in the introduction.

**Authors:** We stated that we are completely open source and do not rely on any non-open source or commercial software (lines 163 to 168). We also stated that we provide a first framework with optimization functions directly linked to the simulations (lines 181 to 186).

**Reviewer 1:** “Large-scale nerve simulations are embarrassingly parallel problems and can greatly reduce computation time. NRV provides multiprocessing capabilities by implementing a message-passing interface (MPI) for Python [35]. Parallel computing is performed independently from the simulation description and the end-user only needs to provide the maximum number of usable cores on the machine; NRV automatically handles job distribution and synchronicity between the processes.” Why is MPI needed for an embarrassingly parallel problem? What information is being passed between processes? Unclear what the impact is, and what is novel compared to existing solutions.

**Authors:** We have rephrased/shortened this statement (lines 187 to 189). We have also added Table 2 (in the Discussion), which shows the benefits of parallelization. MPI is used (among other things) to dispatch NEURON simulation parameters to each computation core, synchronize the cores, and finally merge the results into a single dictionary that is returned to the user. We have chosen to limit the backend information of the framework presented in this document and to prioritize the architectures design and possibilities offered by NRV. Examples of parallel computation are provided in the documentation.

**Reviewer 1:** “Third-party libraries used are fully encapsulated in the NRV framework”. What does this mean? And how does this make these softwares more transparent? What is “transparency”?

**Authors:** We have rephrased and clarified by providing more detail (lines 193 to 197).

**Reviewer 1:** Fmod: “The stimulation current Istim,k is described by a dedicated stimulus-class that defines asynchronous stimulus current and time”. Current is a function of time, and therefore the word asynchronous is not correct.

**Authors:** We have clarified the text (lines 241 to 244). Here asynchronous is used in the sense that stimuli do not depend on a global time reference, are event-based, and do not need to be synchronized with each other. More specifically, operations and combinations of stimuli do not require synchronicity between them.

**Reviewer 1:** Equation 1: confusing notation because V1=I*V2, where V2 (i.e., footprint) is actually V/Amp. I’ve seen this written with “transfer resistance” (Rtrans) where unitary current is delivered, and the resistance between current source and point is calculated using the voltage sensed. Then Vext = Iapplied * Rtrans. From my experience in teaching, this notation is more accessible to people new to the field.

**Authors:** We thank the reviewer for this comment. The notation we used corresponds to the way it is implemented in the code and is mathematically exact and based on the linearity hypothesis. To make it easier for the reader to understand, we have specified the units (line 234) and mentioned the equivalence to a resistive quantity (lines 236 to 238).

**Reviewer 1:** Mechanics of implementation not clear: “Arithmetic operations between Stimulus-objects are defined for the class, thus enabling fully customized arbitrary stimulating waveforms”

**Authors:** This is done by operator overloading in the class definition. This is now mentioned in the text (lines 245 to 248). Examples of combinations are provided in the Appendix (S1).

**Reviewer 1:** Figure 2 caption: word missing? “NRV’s transcription of the physical with dedicated classes that can be combined and are used by the framework to evaluate the extracellular electrical fields”

**Authors:** Thank you for this comment. The word ‘model’ was indeed missing.

**Reviewer 1:** Typo Page 9 “perineum” should be “perineurium”

**Authors:** We corrected this typo, thank you.

**Reviewer 1:** To my knowledge, conductivities for epineurium, endoneurium, and perineurium are not all found in [40]. What are the actual values used for conductivity?

**Authors:** Thank you for this comment. We have clarified the text (lines 270-271) and added a supplementary material (S2) with a table summarizing the material properties implemented in NRV with the corresponding references.

**Reviewer 1:** “However, it restricts the geometry to only one material and the electrode shape is not taken into account, limiting the viability of this approach to model complex experimental or therapeutic setups [43]”. Also nerve geometry and electrode interface are not taken into account

**Authors:** We have rephrased and added that this option severely limits the viability of the model (lines 285-288).

**Reviewer 1:** I do not see a reason for not including the FEM computed potentials to simulate recording? Why limit to analytical? I do not think this is a step-forward as tools for modeling recording from analytical/FEM solution already exist

**Authors:** Yes, we know that there are more complex algorithms to simulate recording. Including such methods has a cost in personnel/time and is not the scientific priority of our lab. This limitation is actually stated in Table 1 in the discussion. As also mentioned in the perspective paragraph, we are currently working on another recording method, i.e. electrode impedance tomography, which will be used in future versions of the framework. Providing NRV to the open-source community also opens up the fork-merge request process for collaborative development and could be used to add such functionality to the framework.

**Reviewer 1:** “In NRV, morphological values in [21] and from [51] are interpolated with cubic or quadratic functions”. Specifically which values? What are the relationships used?

**Authors:** We have added a supplementary material (S3) with original points and fitting curves and equations to clarify this aspect of the framework.

**Reviewer 1:** What are the statistics you used? “Statistics taken from [55–57] have been interpolated and predefined as population-generating functions.”

**Authors:** We have also added a Supplementary Material (S4) with all related information.

**Reviewer 1:** Optimize: Unclear how user actually interfaces with these features

**Authors:** We have rewritten the Optimize section (lines 439 to 472) in hopes of clarifying the design architecture. We only provide a structural view in this section; it is not intended to be a user guide. The "Stimulus Energy Optimization" subsection of the results shows an example of what can be done with NRV's Optimize module. The corresponding commented Python script is available in S11. We have also worked on the online documentation, adding a tutorial and more extensive help on the user-oriented use of these objects. We hope that all these additional resources will help to clarify the use and possibilities of the optimize module.

## Results

**Reviewer 1:** Can’t find contents of Appendix 1, S2 Files

**Authors:** Our apologies for any omission of a file. This should be fixed now.

**Reviewer 1:** What are red dots in Figure 7a and 7b?

**Authors:** Red dots correspond to the relative difference between the two footprints (from COMSOL or FENICS), as indicated in the figure caption.

**Reviewer 1:** Citation instruction to converge for thresholds, not voltage or its 2^nd^ difference: <https://pubmed.ncbi.nlm.nih.gov/24448594/>

**Authors:** We have added this citation (91 and an additional one, reference 92) and commented on it (lines 482-483).

## Discussion

**Reviewer 1:** But NRV *does* depend on external software… e.g., NEURON, Python, optimization packages, etc…

**Authors:** We have clarified and provided more details (lines 781 to 785). The fully open-source approach also allows retrograding to ensure the viability of the results, as requested by Wilkinson et al. (reference number 44).

**Reviewer 1:** You said the NEURON simulations are embarrassingly parallel, so how do you speed up the simulations?

**Authors:** We mainly parallelize calls to NEURON (i.e. multiple NEURON simulations running at the same time). We do not parallelize the NEURON simulation itself, as the NEURON solver's parallelization capabilities are not compatible with NEURON's extracellular mechanism required by NRV (see NEURON documentation https://nrn.readthedocs.io/en/8.0.1/coreneuron/how-to/coreneuron.html). We have added a table (table 2) to the discussion with examples of computational costs on different machines.

## Overall

**Reviewer 1:** Documentation for installation and use is not clear or thorough. What do I change to customize the follow (as example, not exhaustive list):

- Waveform
- Material property
- Fiber model
- Cuff
- Cost function
- Etc

**Authors:** We have worked on the documentation of the code. This now corresponds to version 1.1.0 with the corresponding help (by sphinx compiling the current version on github) and documentation is published on read-the-docs (<https://nrv.readthedocs.io/en/latest/>). We changed the overall structure and added tutorials, guidelines (including the proposed topics and more) and examples in addition to the API documentation generated from docstrings. This work is still in progress and we hope to add more material in the coming weeks as we get feedback from new users.

**Reviewer 1:** Main text is difficult to follow, simultaneously not enough detail to understand how it works (need documentation for users) but also not streamlined enough to appreciate what has been built.

**Authors:** We rewrote several sections of the original document. We have revised our positioning with respect to other existing solutions. We also removed redundancies in this new version, hoping to make it clearer. Work on the documentation has been moved to the public GitHub repository and is published on read-the-docs (<https://nrv.readthedocs.io/en/latest/>). The online help is user-oriented, this current paper cannot replace it, but we hope to focus on the scientific foundations of the framework here.

**Reviewer 2:**

The authors present a research article aiming to develop an open-source framework for in-silico study of peripheral nerve electrical stimulation using multiscale analysis. That

framework could be used by the researcher and clinicians to facilitate the design of different stimulation strategies, considering the possibility of implanting multiple electrodes and testing different types of stimulation waveforms. The authors compared and validated the results obtained from their new open-source Python-based framework with the results based on the most common approaches used in the literature using commercial FEM and data processing software.

The topic in consideration is relevant for the readers of PLOS Computational Biology.

However, the work should be improved before being considered for acceptance in this

journal, possibly taking into consideration the following issues in the revised version.

**Authors:** We would like to thank the reviewers for their careful review. We have addressed all of the comments and detail how we have changed the text below.

**Major comments**

**Reviewer 1:** In the Introduction section (i.e. lines 8-12 or line 28) or in the Results section at line 469, some important articles carried out in the same research area that used the hybrid FEM-Neuron approach are missing:

o C. Schmidt and U. van Rienen, "Adaptive Estimation of the Neural Activation

Extent in Computational Volume Conductor Models of Deep Brain Stimulation,"

in IEEE Transactions on Biomedical Engineering, vol. 65, no. 8, pp. 1828-1839,

Aug. 2018, doi: 10.1109/TBME.2017.2758324.

o Stefano, M., Cordella, F., Gioi, S. M. L., & Zollo, L. (2021, May). Electrical

stimulation of the human median nerve: A comparison between anatomical and

simplified simulation models. In 2021 10th International IEEE/EMBS Conference

on Neural Engineering (NER) (pp. 769-772). IEEE.

o Salkim, E., Shiraz, A., & Demosthenous, A. (2019). Impact of neuroanatomical

variations and electrode orientation on stimulus current in a device for

migraine: A computational study. Journal of neural engineering, 17(1), 016006.

o Stefano, M., Cordella, F., Cozza, G., Ciancio, A. L., & Zollo, L. (2023). Study of

electrical neural stimulation effects using extraneural and intraneural electrodes.

Applied Sciences, 13(10), 6267.

**Authors:** These references have been added to the text and correspond to the numbers 35, 36, 37, and 38, respectively (line 31).

**Reviewer 1:** I suggest to the authors adding the execution time needed for the different steps of a

“test” simulation with fixed properties (i.e. building the geometry, meshing, FEM

solution computing, evaluation of axon fibers activation).

**Authors:** Thanks for the insightful comment. We have written a benchmark script and provided timing results on various machines, from laptops to clusters. This corresponds to Table 2 and is detailed in the discussion section (lines 788 to 791).

**Reviewer 1:** At line 243, PSA is cited. Please, could you specify the conditions for which you can use the PSA?

**Authors:** We mentioned in the text the limitations and a citation (reference number 61, line 259).

**Reviewer 1:** At line 640, why did you use 205 myelinated axons? Please, could you motivate this

choice?

**Authors:** We have described in the text the process used to generate the neural population for the optimization result (lines 645 to 649). It corresponds to the number needed to pack a 200µm in diameter fascicle. This number is arbitrary, but large enough to demonstrate the optimization capabilities of NRV on a non-trivial problem.

**Minor comments**

**Reviewer 1:** At line 241, “ID” can be modified in “1D”.

**Authors:** ID is the correct terminology. We identify each electrode of the model with a unique number so multiple electrodes can be used with individual stimulus. We clarified in the text, changing “ID” with “identifier” (line 254).

**Reviewer 1:** At line 317, the authors cited the no ephaptic coupling hypothesis. Please, could you

add a reference?

**Authors:** We have added reference numbers 34, 46, and 55 to justify this hypothesis in the introduction of the document (line 41).

**Reviewer 1:** In the Section “Perspectives” it is reported at line 792 that “NRV only provides classes and methods to generate parameterized cylindrical nerve and fascicle geometries”. Please, could you add this information also in the section “Our approach: NRV”

**Authors:** We have added this information in the appropriate paragraph (line 166) to avoid any misleading information. Thank you.

**Reviewer 1:** In the caption of Fig. 7, could you cite the research paper that justifies the setting of the thickness of the myelin sheath?

**Authors:** We assume you meant perineurium, not myelin sheath? If not, then the myelin sheath thickness is defined by the MRG model (cited earlier in the paper). While checking this, we also noticed that the chosen perineurium thickness was too low; we changed this value according to the literature (reference 18, perineurium to 3% of fascicle diameter = 24µm) and updated the full figure and results with the corresponding simulations.
